# A holistic methodology for evaluating flood vulnerability, generating flood risk map and conducting detailed flood inundation assessment

**DOI:** 10.1038/s41598-025-13025-z

**Published:** 2025-08-02

**Authors:** Kamalini Devi, Chundi Chenna Reddy, Kandakatla Rahul, Jnana Ranjan Khuntia, Bhabani Shankar Das

**Affiliations:** 1 Department of Civil Engieering, National Institute of Technology Warangal, Warangal, 506004 India; 2 Department of Civil Engieering, Chaitanya Bharathi Institute of Technology, Hyderabad, 500075 Telangana India; 3 Department of Civil Engieering, National Institute of Technology, Patna, 800005 India

**Keywords:** Flood vulnerability, Flood risk map, QGIS, HEC-RAS, Spatial analysis, Unsteady flow analysis, Flood risk assessment, XGBoost, Machine learning technique, Arc-GIS, Flood-prone areas, Hydrology, Natural hazards

## Abstract

Flood risk assessment (FRA) is a process of evaluating potential flood damage by considering vulnerability of exposed elements and consequences of flood events through risk analysis which recommends the mitigation measures to reduce the impact of floods. This flood risk analysis is a technique used to identify and rank the level of flood risk through modeling and spatial analysis. In the present study, Musi River in the Osmansagar basin is taken in to consideration to evaluate the flood risk, which is located at Hyderabad. The input data collected for the study encompasses Hydrological and Meteorological datasets from Gandipet Guage station in Hyderabad, raster grid data for Osmansagar basin along with several indicators data influencing flood vulnerability. The primary research objective is to conduct a quantitative assessment of the Flood vulnerability index (FVI), to develop a comprehensive flood risk map and to evaluate the magnitude of damaging flood parameters, inundated volume and to analyze the regions inundated in the study area. In risk analysis, FVI determines the degree of which an area is susceptible to the negative impact of flood through various influencing indicators, Flood hazard map segregate the regions based on flood risk level through spatial analysis in Arc-GIS. A part of this study includes an integrated methodology for assessing flood inundation using Quantum Geographic Information Systems (QGIS) data modelling for spatial analysis, Hydraulic Engineering Center’s River Analysis System (HEC-RAS) hydraulic modelling for unsteady flow analysis and a machine learning technique i.e. XGBoost, to enhance the accuracy and efficiency of flood risk assessment. Subsequently, inundation map produced using HEC-RAS is superimposed with building footprints to identify vulnerable structures. The results obtained by risk analysis using hydraulic modeling, GIS analysis, and machine learning technique illustrates the flood vulnerability, areas having high flood risk and inundated volume along with predicted flood levels for next 10 years. These findings demonstrate the efficiency of the holistic approach in identifying vulnerability, flood-prone areas and evaluating potential impacts on infrastructure and communities. The outcomes of the study assist the decision-makers to gain valuable insights into flood risk management strategies.

## Introduction

Flooding is an accumulation of water in an area either by direct rainfall or a spill of vast amount of water from water bodies beyond normal limits^23^. Floods signify a typical example of the pulsed type of disturbance in the river^17^. According to World Resource Institute (WRI), India has listed on top among the people affected by river floods around 163 nations in terms of number of people. In India, People from Ladakh, Uttarakhand, Kerala, Maharashtra, Assam and Hyderabad have experienced extreme flash floods for 15 years. As per the India Meteorological Department (IMD), very heavy rainfall occurs during the cloudburst with faster rate and suggests that the rate of rainfall about 100 mm/h^29^. Hyderabad is a city experiencing rapid growth due to population expansion, economic development, and urban sprawl and faces a significant challenge like flooding. People in the Hyderabad city has witnessed flood events during the year 2001, 2003, 2008, 2016 and 2017 in which many under-lying areas were inundated due to heavy rainfall^31^. This sudden rise in flood level due to heavy rains leads to huge devastation, causing significant loss to the structures as well as the people staying in that low lying areas. The combination of climate change, urbanization, and insufficient infrastructure has heightened the city’s vulnerability to flooding events. In response to these pressing issues, this study investigates the feasibility and effectiveness of elevated flood protection measures for buildings in Hyderabad.

By integrating the hydrological modelling, structural analysis, and urban planning strategies, the current goal is to mitigate the negative impacts of floods on residential and commercial structures while promoting sustainable urban development. The rapid urbanization of Hyderabad has dramatically altered its landscape, with extensive development encroaching upon floodplains and wetlands. As the city continues to grow, natural drainage systems are compromised, increasing the risk of flooding. Additionally, the effects of climate change, including altered rainfall patterns and more frequent extreme weather events, further exacerbate Hyderabad’s susceptibility to floods. These factors underscore the urgent need for proactive measures to enhance flood resilience within the city. As Hyderabad navigates the challenges of urbanization and climate change, elevated flood protection measures emerge as a crucial strategy for enhancing flood resilience and promoting sustainable urban development. Majorly the building which are constructed in low lying areas rise to loss of property and lives. By leveraging interdisciplinary approaches and innovative solutions, the adverse impacts of floods on buildings and communities can be mitigated while fostering resilience in the face of evolving climate challenges. Prioritizing collaboration, innovation, and proactive planning is essential to building a safer and more resilient Hyderabad for future generations. Hydrological modelling is crucial for assessing the feasibility and effectiveness of elevated flood protection measures. Through simulations of various flood scenarios, researchers can evaluate how these interventions mitigate flood-related damages and enhance building resilience. Concurrently, structural engineering analyses examine how elevated buildings perform under dynamic flood loads, ensuring their structural integrity and durability. Elevated flood protection measures involve raising the foundations of buildings and implementing flood-resistant construction techniques.

**Fig. 1:**
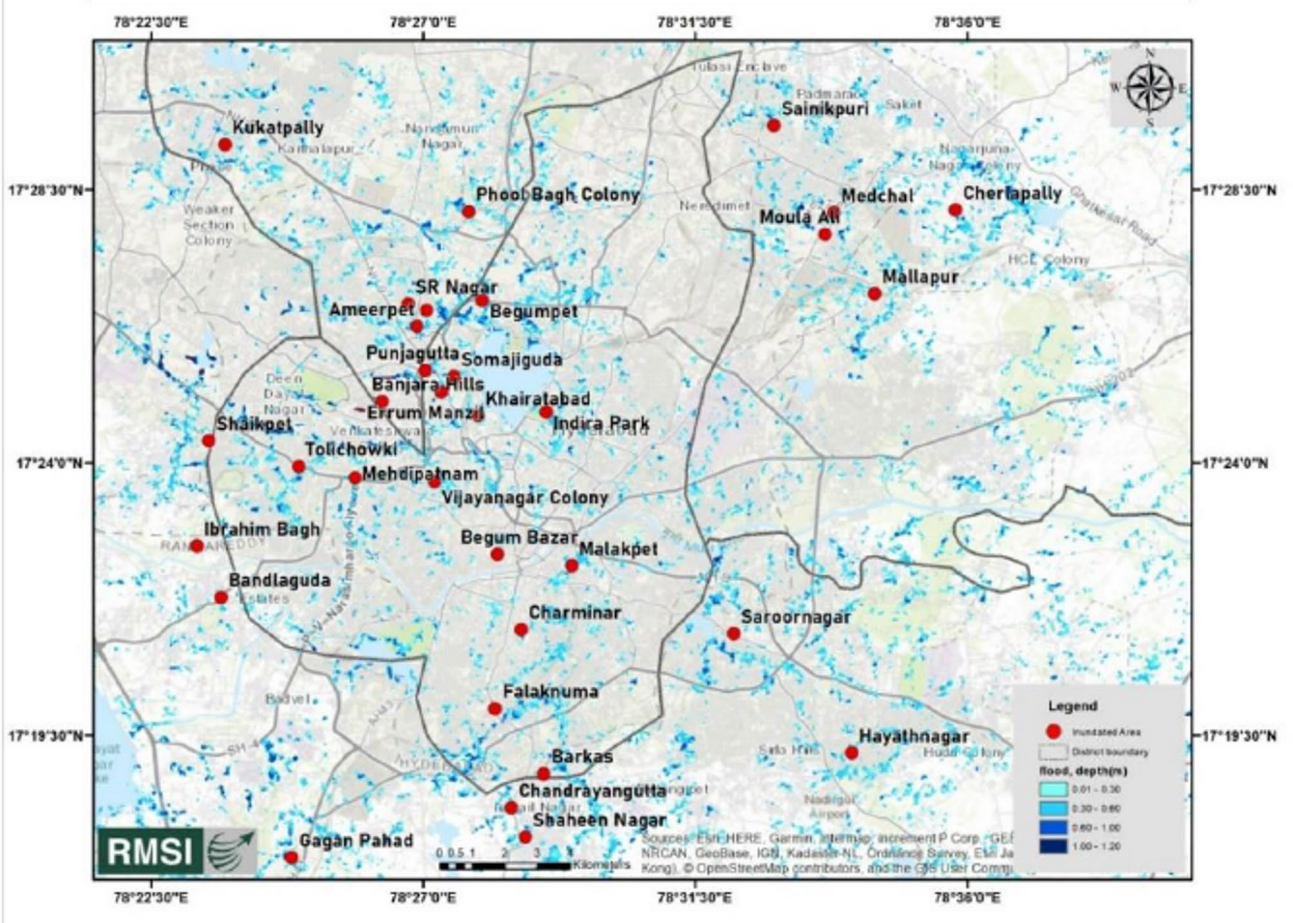
Inundation extent in Greater Hyderabad on 14th October 2020. (Source : RMSI).

**Fig. 2:**
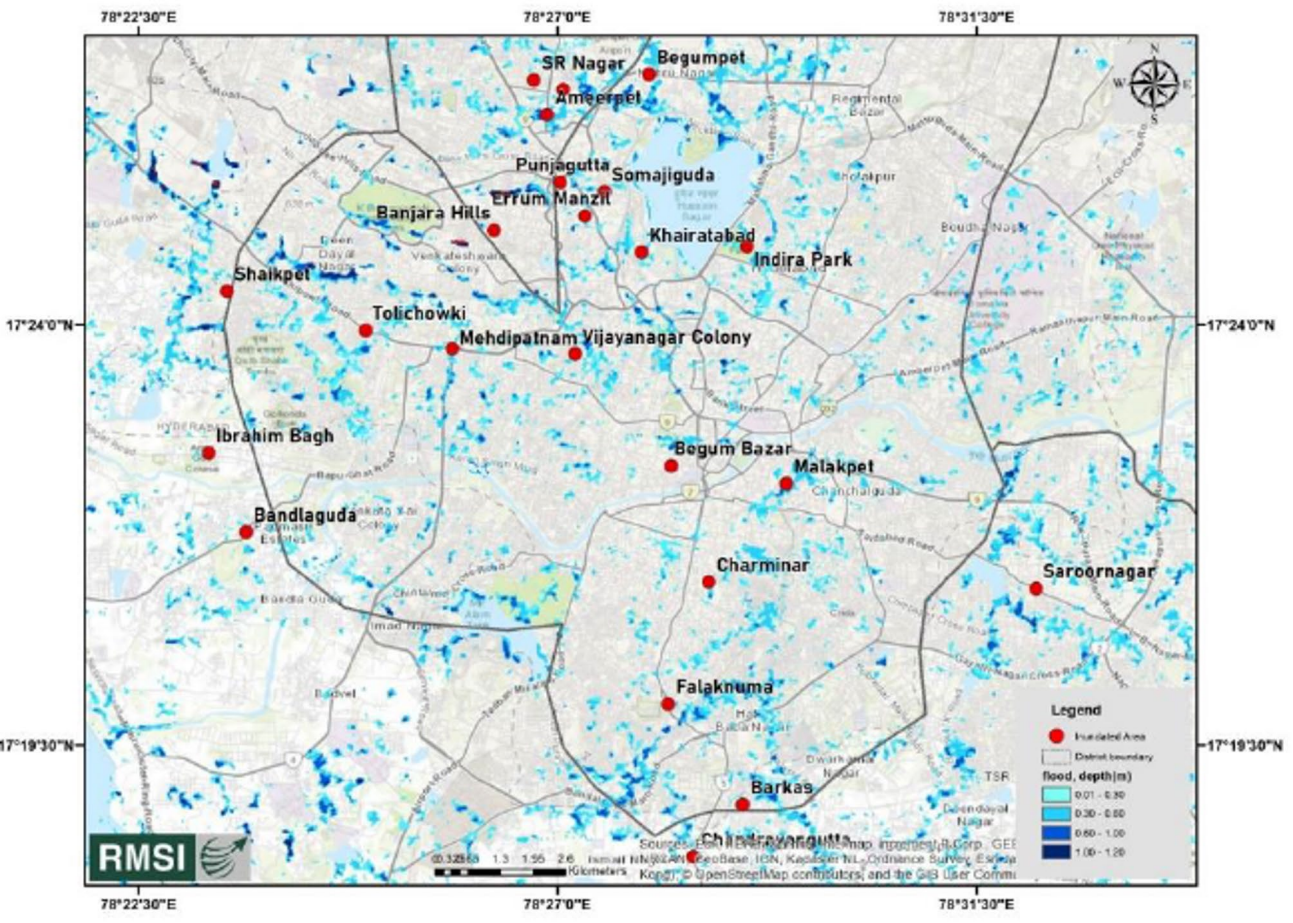
Inundation extent in Hyderabad on 14th October 2020. (Source : RMSI).

This proactive approach aims to minimize structural damage, protect residents, and strengthen built environments against flooding. By elevating buildings above potential flood levels, vulnerability can be reduced, and critical assets safeguarded from flood-related harm. The main focus has been imparted to a part of Hyderabad (Narsingi and Gandipet area), after experiencing vicious flood scenarios since last 5 years. Figures 1 and 2 represents inundation extent in Greater Hyderabad and Hyderabad regions due to rainfall on 14th October 2020, analysed by Risk Management Solutions, Inc (RMSI)^26^.

Rangari et al.^24^ proposed an innovative approach utilizing HEC-RAS 2D integrated with GIS to evaluate inundation risks in Hyderabad by delineating vulnerable zones and categorizing them according to flood risk severity. Similarly, Madhuri et al.^19^ delved into urban flood risk analysis, emphasizing the necessity of accounting for climate change variables and devising adaptive strategies to bolster building resilience. In a departure from conventional techniques, Madhuri et al.^20^ delved into the realm of machine learning for flood susceptibility assessment within the Greater Hyderabad Municipal Corporation region through shedding light on the efficacy of algorithms like XGBoost in classifying flood-prone areas. Building upon this groundwork, Madhuri et al.^21^ introduce the innovative concept of Flood-susceptibility-based building risk (FSBR), amalgamating XGBoost and HEC-RAS 2D to comprehensively comprehend flood impacts amid changing climatic conditions. Furthermore, Venkatesan and Mahindrakar^33^ proposed a novel methodology for flood forecasting by employing Extreme Gradient Boosting (XGBoost), showcasing its superiority in short-term prediction accuracy compared to conventional models. Ma et al.^18^ focused on flash flood risk assessment through an XGBoost-based approach that surpasses traditional methods, thereby offering reliable risk assessments crucial for flash flood mitigation strategies in Yunnan Province, China. This collective body of research underscores the imperative of harnessing advanced technologies and interdisciplinary methodologies to effectively address and mitigate flood risks in urban landscapes, particularly amidst the challenges posed by climate change.

In previous years, researchers have extensively investigated methods to tackle the challenges presented by flash floods, particularly in regions like the Bâsca Chiojdului River Basin in Romania. Abedi et al.^1^ conducted a thorough study utilizing classification and regression tree (CART) methodology and ensemble models such as random forest (RF), boosted regression trees (BRT), and extreme gradient boosting (XGBoost). Their research aimed to delineate areas susceptible to flash floods by analyzing various factors including slope, land use and land cover (LULC), and aspect. The results of their study, published that year highlighted the slope as the primary factor triggering flash flood occurrences, with the RF model proving most effective in mapping flash-flood susceptibility. Sanders et al.^28^ focused on a separate investigation for developing a Data-Driven Flood Alert System (FAS) using extreme gradient boosting (XGBoost) to forecast flood stages. Their study, centered on a flood-prone watershed in Houston, Texas, aimed to provide accurate predictions of gauge stage levels at short intervals. They demonstrated the effectiveness of the FAS in predicting flood arrival times, without relying on external starting triggers, thereby enhancing flood warning and mitigation practices. Additionally, Ariyani et al.^3^ conducted a flood hazard mapping study in the Bangko and Masjid watersheds in Riau, Indonesia, utilizing Quantum GIS (QGIS) spatial analysis. Their research involved analyzing factors such as slope, land cover, elevation, rainfall, and soil type to assess flood risk and vulnerability. Their findings underscored the high vulnerability to flooding in both watersheds, emphasizing the need for clear flood hazard maps to inform mitigation strategies effectively. Ahmed et al.^2^ explored urban flooding in Hyderabad, highlighting the distinct differences between urban and rural flooding. Their research emphasized the substantial increase in flood peaks and volumes due to urbanization, posing immediate risks to critical infrastructure and economic activities. They advocated for a holistic approach to stormwater management to ensure sustainable urban development.

Flood assessment, prediction, and mitigation requires combined meteorological and morphometric data with 2D HEC-RAS, and QGIS techniques^10^. These studies collectively underscore the importance of employing advanced methodologies, such as machine learning models and spatial analysis, to address the complexities of flood susceptibility and management in various geographical contexts. The flood monitoring has been done using numerical methods and AI-ML technique. While numerous studies have analysed the impact of flood using maching learning techniques, numerical methods and GIS, there is a significant gap in integrating all the above approaches to enhance the efficiency of accuracy of flood risk assesment. As future conditions are expected to worsen, it is crucial to implement the integrated flood risk management strategies that incorporate flood modelling^13^.The primary research objective is to conduct a quantitative assessment of the FVI within the study area, to develop a comprehensive flood risk map and to evaluate the magnitude of damaging flood parameters such as depth, velocity and inundated volume and also to analyze the areas inundated by overlay analysis in the study area.

### Novelty of the study

The novelty of the present study is an integrated approach for comprehensive flood risk assessment in the Musi River basin, Hyderabad. Unlike conventional methods, this study combines Geographic Information System (GIS)-based spatial analysis, HEC-RAS hydraulic modeling for unsteady flow simulations, and machine learning algorithms to enhance the precision and efficiency of flood susceptibility mapping and risk prediction. The key innovative aspect involved in the study is to overlay HEC-RAS-derived flood inundation maps with building footprint layers in QGIS that enables a direct evaluation of structural vulnerability to flooding at a localized scale. Furthermore, the use of machine learning techniques forecast flood scenarios over a 10-year period which provides a future oriented assessment that is rarely addressed in similar studies. The flood susceptibility analysis incorporates a multi parameter assessment that includes topographical parameters such as elevation, slope, land use, and land cover and also integrates hydrological characteristics like rainfall and sub-basin drainage density. The FVI is computed through normalization of multiple influencing indicators, offering a quantitative measure of flood impact potential.

This integrated methodology enhances the accuracy of flood risk modeling by providing practical insights for decision makers through identifying vulnerable regions, structures and recommends mitigation measures. The holistic integration of machine learning techniques, GIS tools, and hydraulic simulation represents a novel framework for urban flood risk analysis in rapidly developing regions like Hyderabad.

The scientific merit of this study lies in its novel integration of diverse analytical tools, its validation with real time data, and its practical implications for urban flood risk management in data-scarce environments.

## Methodology

**Fig. 3:**
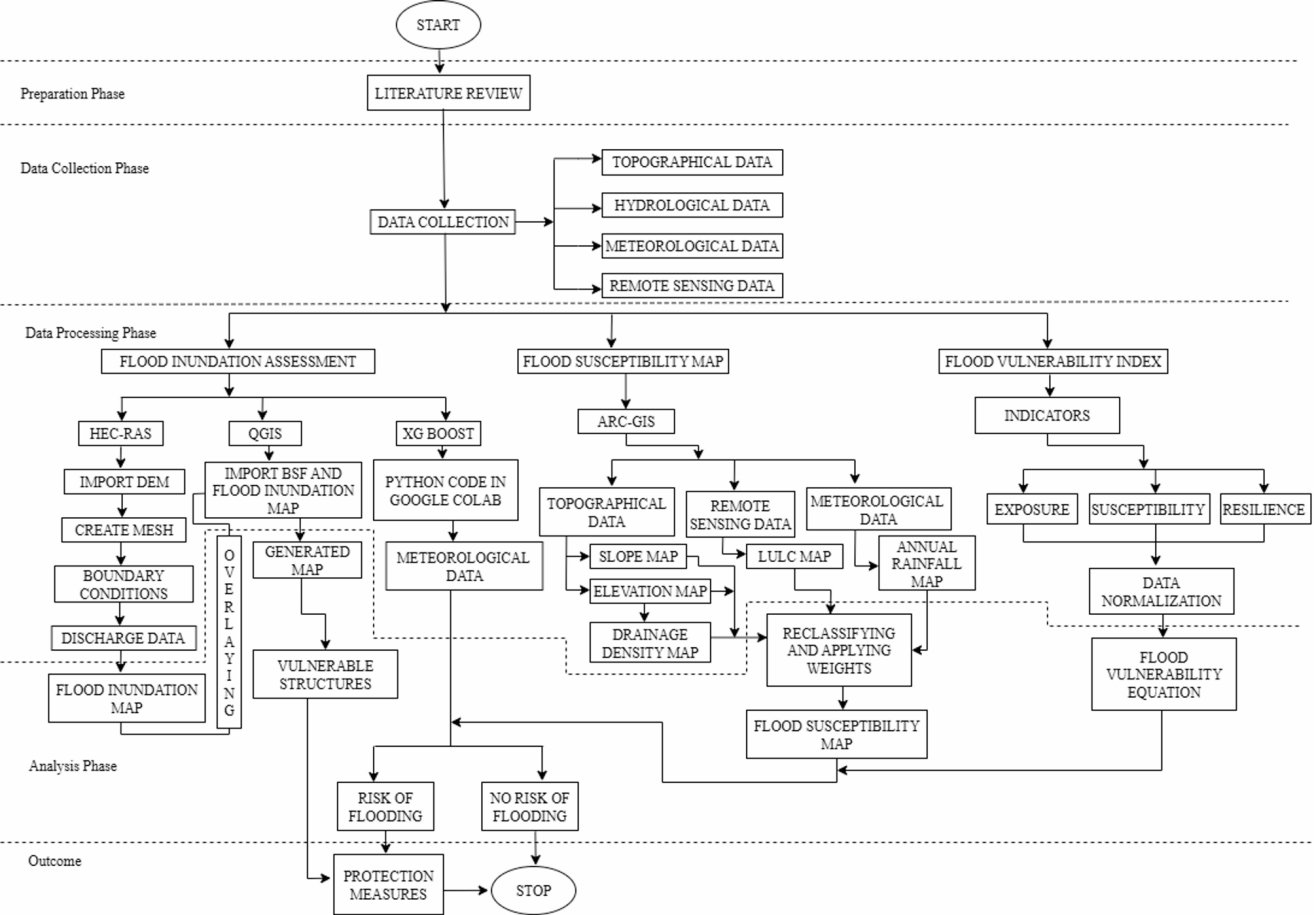
Methodology flowchart.

The methodology adopted in this study is structured into four primary phases such as Preparation, Data Collection, Data Processing, and Analysis phase as illustrated in Fig. 3. The study commenced with a comprehensive literature review to identify relevant methods, tools, and data requirements for flood risk assessment.

Figure 3 demonstrates the methodology along with the steps involved in the study. Each component from this methodology is further explained in detail. Use of this recognized and validated tools (HEC-RAS, QGIS, ArcGIS) enhances reliability^10^.

### Flood inundation assessment

Flood simulation is performed using HEC-RAS with unsteady flow analysis. Input data such as DEMs, boundary conditions, and discharge values were used to create mesh layers and simulate flood extent analysis^24^. The resulting flood inundation maps were exported to QGIS, where they were overlaid with building footprint layers to identify vulnerable structures. Key input data such as elevation models and building shapefiles are obtained from NASA Earth Data and OpenStreetMap.

### Hydrologic engineering Center—River analysis system (HEC-RAS)

HEC-RAS is a software program used to perform one-dimensional and two-dimensional steady and unsteady flow simulations, sediment transport computations, and water quality modelling. The steady and unsteady flow components of this software are capable of modelling supercritical, subcritical and mixed flow regime water surface profiles^16^. It includes HEC-RAS Mapper for spatial data integration and floodplain mapping. The software is widely used in flood risk assessment, hydraulic structure design, and river management. In this study, HEC-RAS software is employed to generate flood inundation maps using elevation and discharge data from the selected study area.

### Quantum geographic information system (QGIS)

QGIS is an open-source GIS software used for viewing, editing, and analyzing geospatial data. It supports multiple data formats and projections and runs on macOS, Windows, and Linux^15^. It is widely used in environmental science, urban planning, and resource management. QGIS provides tools for mapping, spatial analysis, and data visualization through its core features and plugins. In this study, QGIS software is used to import building footprint layers and overlay it with the flood inundation map which is extracted from HEC-RAS to identify inundated areas.

Building exposure is assessed by overlaying the flood inundation extent generated from HEC-RAS simulations onto the building footprint shapefile using QGIS^19^. The shapefile, obtained from OpenStreetMap, contains spatial polygons representing individual building structures within the study area. Each building’s spatial intersection with inundation zones were analyzed to determine exposure. In the present study, exposure is then quantified on the basis of count, which describes the number of buildings intersecting flood inundation map generated from HEC-RAS.

### XG boost

XGBoost (Extreme Gradient Boosting) is a high-performance machine learning algorithm based on the gradient boosting framework^20^. It builds decision trees sequentially, with each tree correcting the errors of the previous one. Commonly used in classification, regression, and ranking tasks. XGBoost is known for its speed, scalability, and accuracy in handling large datasets. Compared to simpler models like Decision Trees (DT) and Random Forests (RF), XGBoost offers enhanced predictive performance by optimizing multiple decision trees in parallel and minimizing residual errors. In this study, the XGBoost library is used to predict flood levels over the next 10 years using 50 years of historical data.

The hyperparameters for the XGBoost regression model were selected using a grid search with 5-fold cross-validation to optimize prediction accuracy and control overfitting. Each parameter is then tuned carefully within chosen range based on the characteristics of the rainfall dataset. The hyperparameters used in the code for prediction of flood levels is given below.

#### n_estimators (20, 40, 60)

This defines the number of boosting rounds. A range of lower to moderate values is then tested to balance training time and prevent overfitting, especially on smaller datasets. A moderate value ensures that the ensemble has sufficient complexity without becoming overly prone to noise.

#### max_depth (3, 5, 7)

Controls the complexity of individual trees. Shallow trees (depth 3–5) are often more generalizable, reducing the risk of overfitting, while still capturing meaningful interactions between features such as humidity, wind, and temperature.

#### learning_rate (0.1, 0.2, 0.3)

Also known as shrinkage, this controls how much each tree contributes to the overall model. Lower values (e.g., 0.1) typically yield more stable models, while higher values (e.g., 0.3) speed up convergence. This range is tested to balance learning speed and model generalizability.

#### Subsample (0.8, 0.9, 1.0)

Introduces randomness by sampling a fraction of the training data for each tree, reducing variance and preventing overfitting. The values chosen (80–100%) help maintain model stability while adding sufficient randomness to promote generalization.

#### min_child_weight (1, 3, 5)

This parameter controls the minimum sum of instance weights in a child node. Higher values make the algorithm more conservative. Testing this parameter ensures that splits are not made on nodes with low instance counts, thus improving robustness against noise.

#### objective=’reg: squarederror’

As the task involves predicting continuous rainfall/flood levels, the squared error loss function is chosen for regression consistency.

### Flood susceptibility map

#### Arc-GIS

ArcGIS facilitates the compilation, editing, and spatial analysis of geospatial datasets while enabling the effective visualization and interpretation of attribute data. In this study, ArcMap is employed to analyze key raster layers such as slope, rainfall, drainage density, land use/land cover (LULC), and elevation for the Osmansagar basin. These layers were subsequently used to generate a flood susceptibility map through weighted overlay analysis in ArcGIS. The integration of GIS with remote sensing technologies offers an efficient approach to flood risk assessment by transforming large datasets into spatially distributed maps that reveal trends, spatial relationships, and potential inundation zones^12^.

### Sensitivity analysis

For evaluating the robustness of flood susceptibility map, a sensitivity analysis is performed to investigate the extent of flood risk per flood risk zone by varying weights of influencing parameters such as Annual rainfall, Land use and Land cover (LULC), Slope, Elevation and Drainage density.

Each parameter weight is individually increased and decreased by 10% to examine the effect on flood risk zone classification. The modified scenarios include high and low weighing conditions for each factor. The high weighing factor indicates the 10% increase in baseline weight of the parameter and low weighing factor indicates the 10% deduction of baseline weight of the parameter. During the modification of weighing factor, the deviation of 10% weight for a particular parameter is equally added or reduced for the remaining parameters weight to maintain the sum of weights value to 1.

### Flood vulnerability index (FVI)

**Fig. 4:**
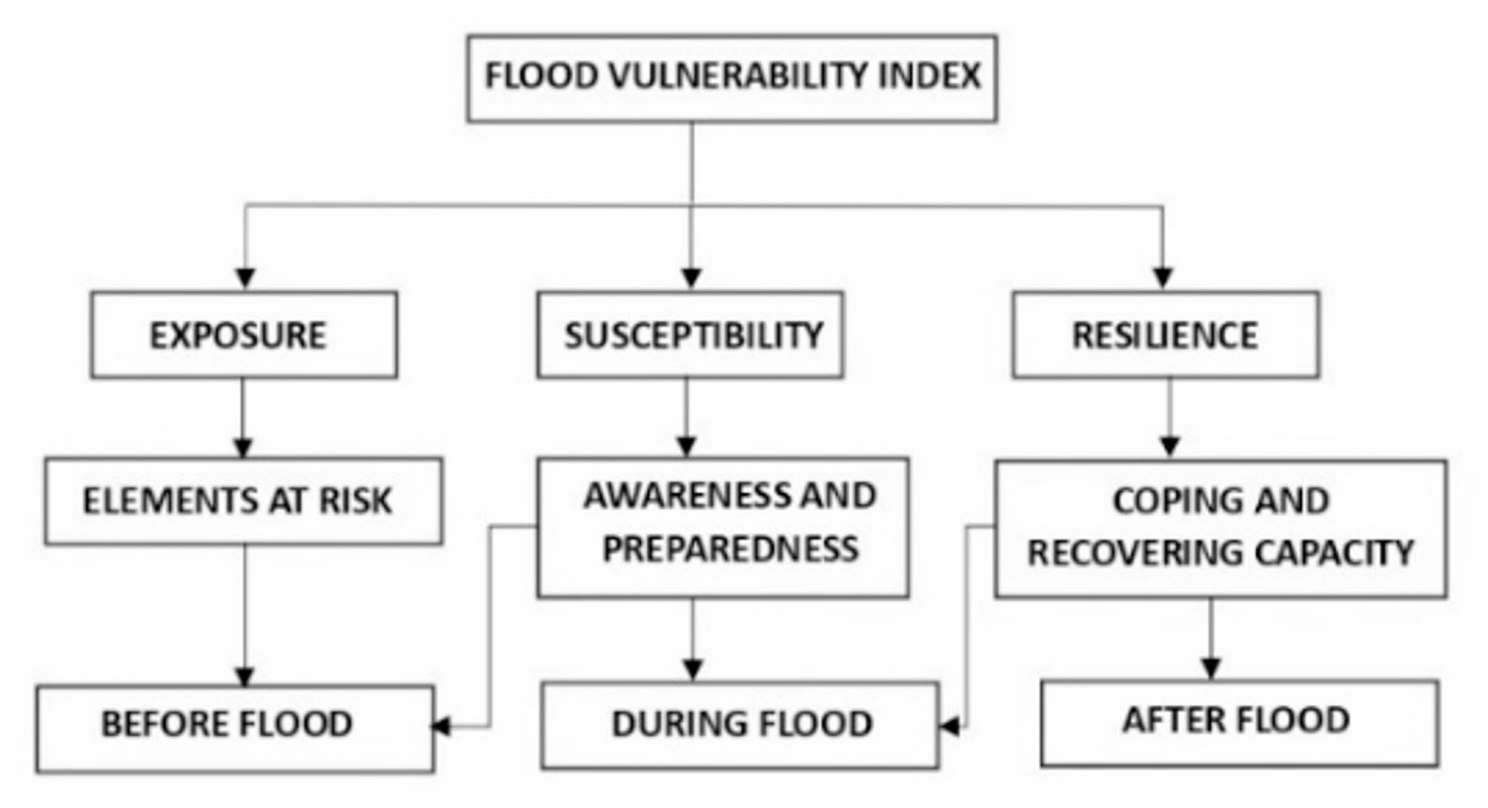
Factors of Vulnerability.

FVI is a tool that measures the degree or extent of occurrence of floods under certain conditions. The FVI is influenced by several factors such as exposure, susceptibility and Resilience^4^. In the present study, normalization technique is used for 11 influencing indicators to calculate exposure, susceptibility and resilience. Figure 4 demonstrates the factors of vulnerability considered before, during and flood occurrence.

### Selection of input parameters

The causes of Hyderabad floods, 2020 are the key reason behind selecting the input parameters for calculating exposure in FVI. Risk Management Solutions, Inc (RMSI) reported that, “Hyderabad has experienced a worst rainfall in 117 years with 19.2 cm of average rainfall on October 13th 2020”^26^. The excessive rainfall triggered the sudden rise of water level and discharge in the Musi River and inundated the low elevated areas in Hyderabad.

As per the Telangana state statistical Abstract 2021, Hyderabad having highest population density including most built-up areas among all districts of Telangana which are highly prone to floods. Therefore, the above input parameters are selected for calculating susceptibility and finally for Resilience, inadequacy of Automatic warning stations (AWS) and dams to safeguard the downstream areas during the scenarios like Hyderabad flood 2020 is the major purpose of selecting the above input parameters.

**Exposure-** The impact of flood for livelihood consists of humans and physical objects like infrastructure, goods and agricultural fields which are identified before flood. Table 1 shows the indicators considered for calculating Exposure factor of vulnerability.

**Table 1:** Indicators for Exposure.

Hydrological	Water level, Discharge and drainage density
Climatological	Monthly Rainfall data
Topographical	Elevation and slope

**Susceptibility-**It relates to the awareness and preparedness of the people those who will affect by the flood with respect to the degree of risk they live in the flood prone areas. This factor safeguards the victims before and during the flood. Table 2 describes the susceptibility indicators considered for the study.

**Table 2:** Indicators for susceptibility.

Social	Population Density, Literacy Rate (%)
Infrastructure	Number of building prone to flood

**Resilience-** It is the capacity of a system to resist the deviations caused in the system due to the influence of flood in maintaining the significant levels of efficiency in social, economic and physical objects after the flood. Table 3 shows the resilience indicators considered for this study.

**Table 3:** Indicators for resilience.

Governance	AWS
Infrastructure	Number of Dams

### Vulnerability equation

Vulnerability = Exposure + Susceptibility – Resilience.

Considering real time data of above-mentioned indicators enhance the accuracy of FVI computation.

### Sensitivity analysis

To evaluate the robustness and influence of each indicator on the FVI, a Leave-One-Out (LOO) sensitivity analysis is performed. This process involves removing one indicator at a time from the original set of indicators, and recalculating the FVI using the remaining indicators. This process is repeated for all indicators across the three FVI components such as exposure, susceptibility, and resilience. The variation in the FVI score after removing each indicator is then compared with the original (base) FVI computed using all indicators. This method helps in identifying the most dominant indicators contributing to flood vulnerability and supports more targeted decision-making in disaster risk reduction planning. All values used in the analysis were normalized prior to index calculation to ensure comparability.

### Risk estimation procedure

In this study, flood risk is estimated by integrating three core components like Hazard, Exposure, and Vulnerability.

#### Hazard estimation

To generate the flood hazard map, individual spatial layers including slope, elevation, drainage density, LULC, and rainfall maps were derived using ArcGIS tools^3^. These layers were then reclassified based on defined risk levels. Subsequently, using a combination of subject matter expertise and existing literature, weights were assigned to each layer reflecting its relative influence on flood susceptibility. A weighted overlay analysis is performed in ArcGIS to integrate the layers and produce the final flood hazard map. Additionally, a machine learning-based dynamic hazard prediction is conducted using the XGBoost model to estimate future flood levels for the next decade. This predictive model strengthens the hazard assessment by incorporating temporal flood behaviour based on historical meteorological data.

#### Exposure estimation

Exposure is evaluated by overlaying the HEC-RAS generated inundation map with the building footprint layer in QGIS. This building’s spatial intersection with inundation zones were analyzed to determine exposure. Counting the number of buildings located within the inundated area, indicating built-up areas exposed to flood risk. This quantification helps estimate the extent of potential impact on infrastructure.

#### Vulnerability assessment

For the vulnerability assessment, the selected indicators were grouped into exposure, susceptibility, and resilience categories^4^. Each indicator underwent normalization to ensure uniform scale and comparability. The normalized values for each category were then aggregated, and the resulting values for exposure, susceptibility, and resilience fed into the FVI equation to compute the final vulnerability scores.

### Uncertainty quantification

#### Hazard uncertainty

Related to DEM resolution, hydrological input precision, and the assumptions in GIS-based overlay methods. Comes from the subjective weighting of indicators. To reduce bias, literature-based weight selection and expert knowledge were applied. For future flood level prediction using XGBoost, RMSE values were computed, and model performance is then evaluated to capture uncertainty in predictive reliability.

#### Exposure uncertainty

May arise geometry errors in vector data in building footprint layers. This error is corrected by fix geometries tool in QGIS.

#### Vulnerability uncertainty

Errors in validation of the FVI through comparison with actual flood damage data (e.g., economic loss, displacement statistics) is limited or unavailable, which hinders quantitative uncertainty estimation through ROC curves, correlation analysis, or residual errors.

### Study area

**Fig. 5:**
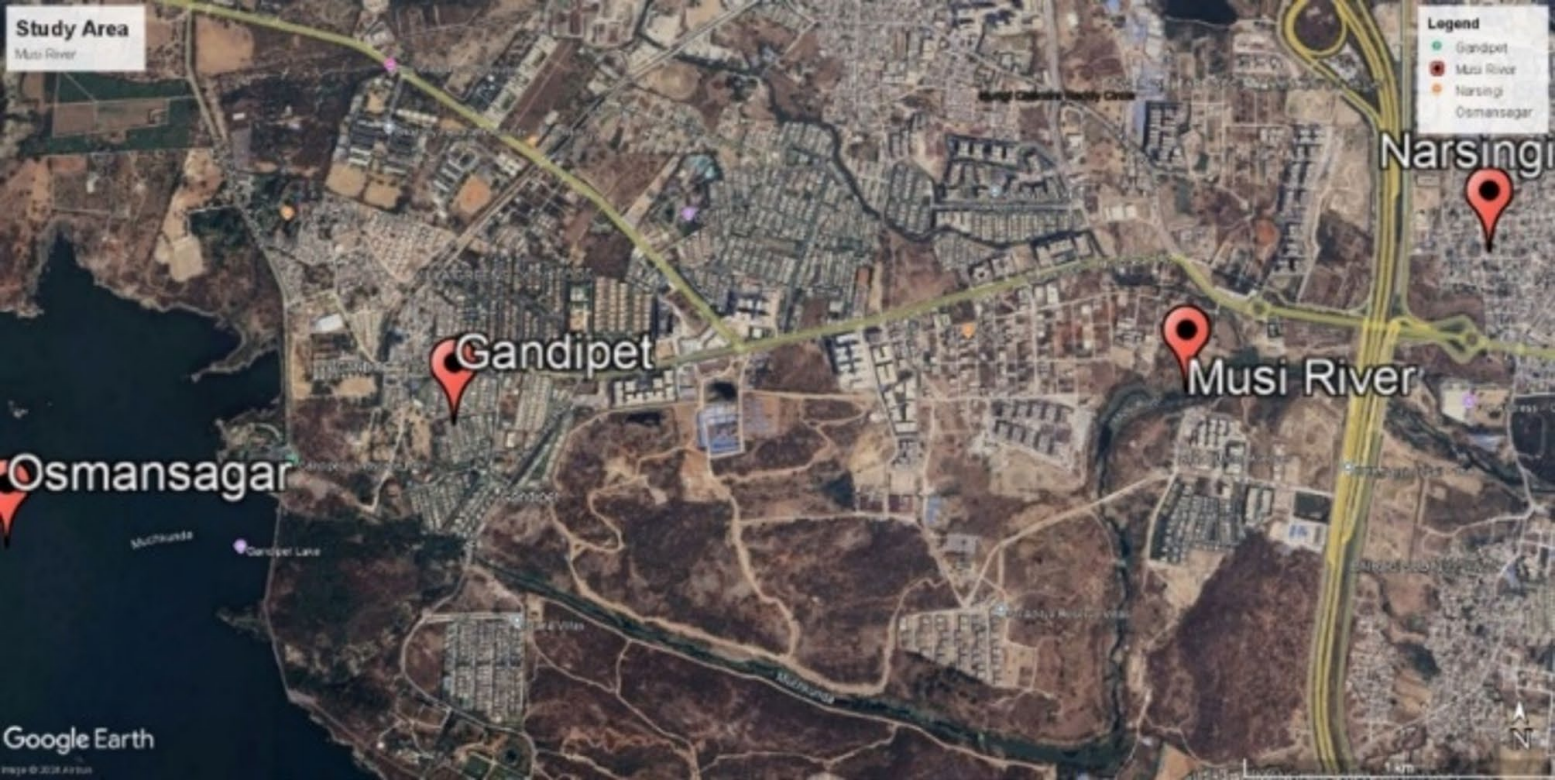
Satellite view of River Musi from Google Earth pro software (version 7.3.6.10201; available at: https://www.google.com/intl/en_in/earth/about/versions/).

The selected study area is River Musi located between Narsingi and Gandipet at latitude and longitude ((17.377966,78.327893),(17.384737,78.349514)). The satellite image of study area is extracted from Google earth pro software as shown in Fig. 5.

### Data collection

#### Flood inundation assessment

##### Digital elevation model (DEM)

NASA Earthdata is available from Earth Observing System Data and Information System (EOSDIS) at Distributed Active Archive Centers (DAACs) that holds the data in a cloud platform. This EOSDIS allows user to access the earth data such as temperature, elevation plot, surface reflectance, surface minerology etc. for a selected area. In the present study NASA Earth data is used to download the Elevation data of study area by ASTER Global Digital Elevation Model V003. This DEM is further used to model the 2D flow in the HEC-RAS Software.

**Fig. 6:**
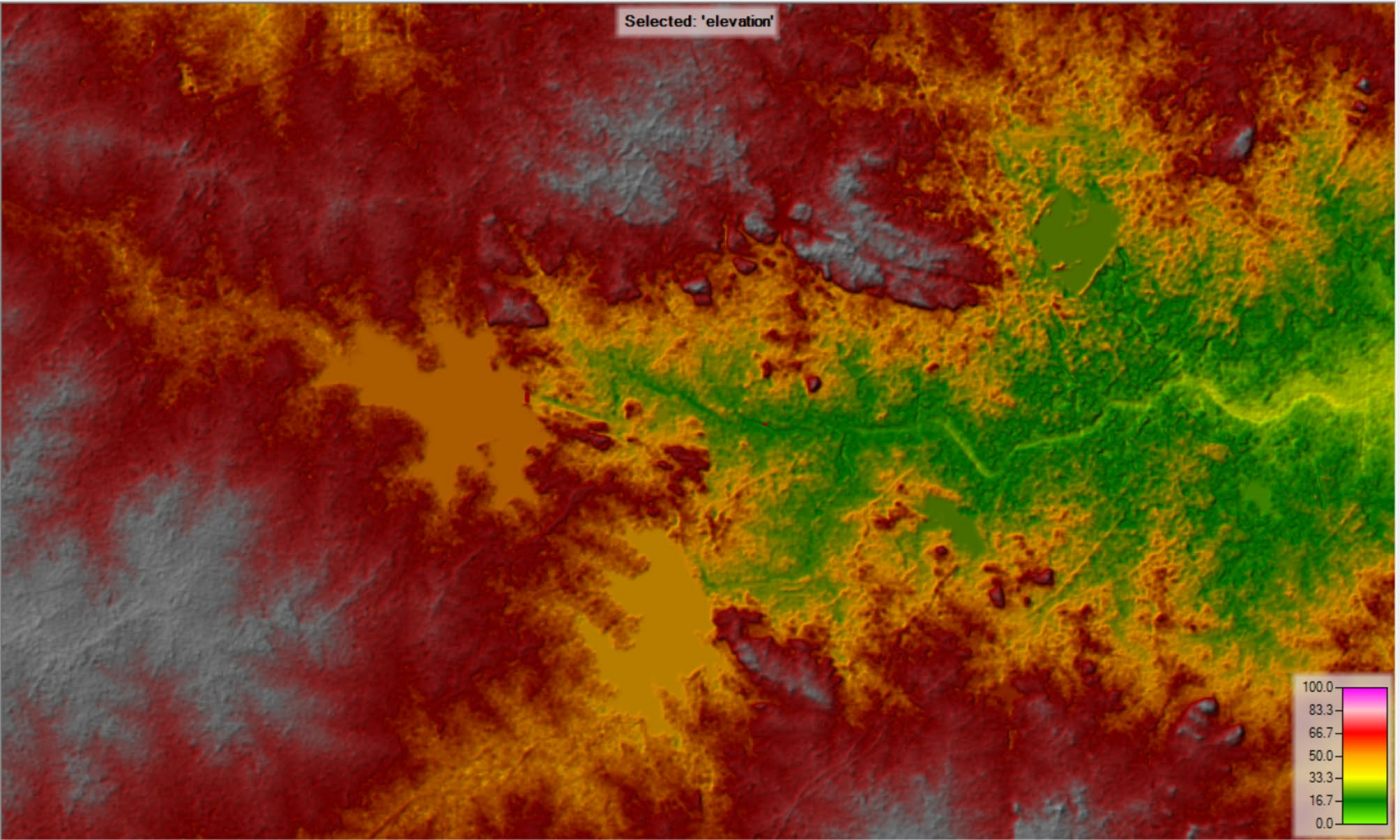
DEM of Study area from HEC-RAS software (version 5.0.7; Available at: https://www.hec.usace.army.mil/software/hec-ras/download.aspx).

Figure 6 represents the elevation data of selected study area. Differnent colours represents different elevation levels that are increasing from light green to grey colour where light green represents lowest elevation and grey colour represents highest elevation.

##### Uncertainty in DEM

The occurrence of long-range noise in DEMs creates a critical limitation in the analysis of DEM precision, and directly affects the uncertainty propagation^11^. Uncertainty refers to our lack of knowledge about this error. Sources of possible error in DEM data sets include^5^:

- Errors can arise from outdated data, sparse observational density, or issues with spatial sampling.
- Measurement errors may include positional inaccuracy, data entry mistakes, or observer bias.
- Processing errors can stem from numerical issues in computational work, interpolation problems, or challenges with classification and generalization.

The uncertainity or inaccuracies have been observed in many DEMs that are generated from air- and space-borne sensors.

##### Building shapefile layer

Building shapefiles play a crucial role in urban planning, disaster management, infrastructure development, and environmental analysis. They provide spatial details about the distribution, size, and features of buildings within specific regions, aiding in decision-making processes and spatial analysis in various fields. The building shape file is downloaded from open source street map for case study area between Narsingi and Gandipet. This shapefile layers consisting of building, roads, water and railways etc. Currently the study focuses on the building shape file layer to observe the area of building inundated to the flood.

**Fig. 7:**
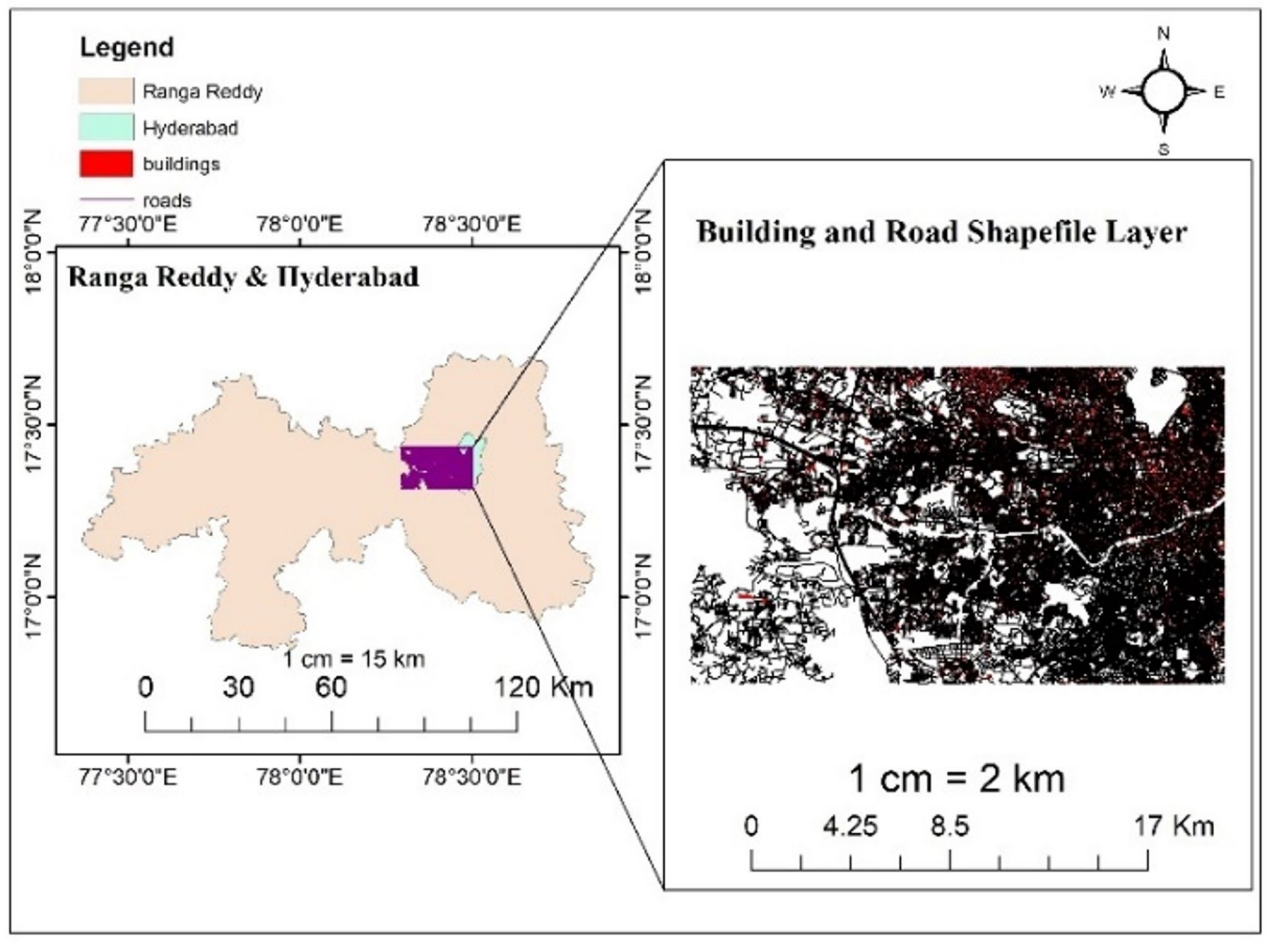
Building and Road Shape file layer of Study area from Arc-Map software (version 10.3.1; Available at: https://www.esri.com/en-us/arcgis/products/arcgis-desktop/overview).

Figure 7 demonstrates a building and road shapefile layer which is a file format utilized in QGIS and ARC-GIS to store geometric data related to buildings and roads. It comprises outlines or footprints of buildings represented as polygons or multipolygons and roads represented as lines. These files often include attributes such as building height, land use, or other pertinent data associated with each building. The road shapefile layer consists of Road number, length, width and type of pavement etc.

In this study, this open access web-based platform is used to extract shapefile layers of study area.

#### Discharge data

The Discharge data of river Musi has been collected from HMWSSB (Hyderabad Metropolitan Water Supply and Sewerage Board) which refers the amount of water flowing through the river at a specific location and time. This data helps scientists, engineers, and policymakers in understanding the behavior of rivers, predict floods, manage water resources, and assess the health of aquatic ecosystems.

By analyzing discharge data, we can make decisions about water management and ensure the safety and sustainability of communities living near rivers and streams.The discharge data which is received from HMWSSB contains gauge, water level and discharge values on hourly basis as given in Table 4. This discharge data is given as input in HECRAS at upstream which results to give flood inundation map. The below table shows the discharge data collected from Gandipet gauge station for unsteady flow simulation in HEC-RAS.

**Table 4:** Discharge and water level data of musi river from Gandipet Guage station.

Date	Time	Gauge	Water Level (ft)	Discharge (cf/s)
13-10-2020	12:00:00 AM	0.26	55.26	706.65
13-10-2020	1:00:00 AM	0.26	55.26	765.3
13-10-2020	2:00:00 AM	0.26	55.26	798.3
13-10-2020	3:00:00 AM	0.26	55.26	832.4
13-10-2020	4:00:00 AM	0.27	55.27	898.4
13-10-2020	5:00:00 AM	0.27	55.27	903.8
13-10-2020	6:00:00 AM	0.26	55.26	911.9
13-10-2020	7:00:00 AM	0.26	55.26	920.55
13-10-2020	8:00:00 AM	0.28	55.28	977.1
13-10-2020	9:00:00 AM	0.26	55.26	1005.6
13-10-2020	10:00:00 AM	0.25	55.25	1054.77
13-10-2020	11:00:00 AM	0.2	55.2	1208.54
13-10-2020	12:00:00 PM	0.28	55.28	1379.5
13-10-2020	1:00:00 PM	0.31	55.31	1587.7
13-10-2020	2:00:00 PM	0.33	55.33	1652.29
13-10-2020	3:00:00 PM	0.56	55.56	1603.9
13-10-2020	4:00:00 PM	0.56	55.56	1543.02
13-10-2020	5:00:00 PM	0.56	55.56	1435.7
13-10-2020	6:00:00 PM	0.55	55.55	1209.66
13-10-2020	7:00:00 PM	0.52	55.52	1088.5
13-10-2020	8:00:00 PM	0.52	55.52	999.5
13-10-2020	9:00:00 PM	0.52	55.52	965.5
13-10-2020	10:00:00 PM	0.26	55.26	894.7
13-10-2020	11:00:00 PM	0.28	55.28	798
14-10-2020	12:00:00 AM	0.28	55.28	752.7
14-10-2020	1:00:00 AM	0.28	55.28	708.18
14-10-2020	2:00:00 AM	0.29	55.29	667.7
14-10-2020	3:00:00 AM	0.29	55.29	620.2

#### Upstream boundary condition

**Fig. 8:**
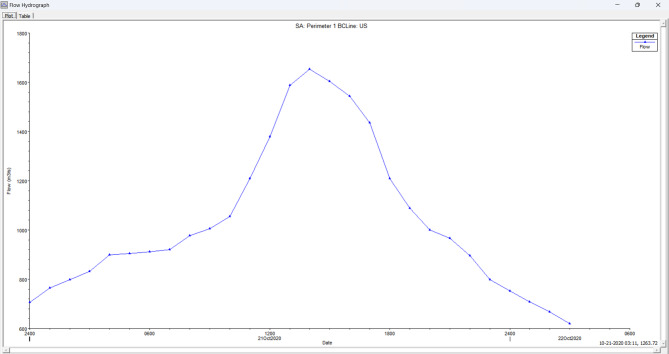
Inflow Hydrograph of Musi River from HEC-RAS software (version 5.0.7; Available at: https://www.hec.usace.army.mil/software/hec-ras/download.aspx).

In HEC-RAS software, during unsteady flow simulation, the Inflow flood hydrograph is used as an upstream boundary for any hydrodynamic study. Figure 8 shows the inflow hydrograph of Musi River plotted by the discharge values collected from Gandipet gauge station. This Inflow Hydrograph used as an upstream boundary condition.

#### Downstream boundary condition

In the present study, the Normal depth (friction slope = 0.05) has been used as the downstream boundary condition for the unsteady flow simulation to the selected study area. After the simulation, Flood Inundation map is generated in HEC RAS to determine the area submerged due to overbank flow conditions around Musi River.

### 2D mesh and cell size

HEC-RAS software supports both the unstructured and structured computational meshes. Structured meshes are considered the same as unstructured ones. The software assumes cells that are orthogonal to each other, with faces perpendicular to a line linking their centers. Computational cells can be triangles, squares, rectangles, or even five and six-sided elements. However, the model only allows for up to eight sides. A 2D mesh has been created to assess flood inundation in the downstream area, comprising a total of 3,207 cells. The maximum cell size is 19,778.33 m^2^, the minimum cell size is 8,614.61 m^2^, and the average cell size is 10,197.35 m^2^.

The HEC‑RAS 2D User’s Manual emphasizes that cell sizes should be considered as per the terrain variability and flow complexity. The smaller cells are used in the locations where rapid changes occur in water surface elevation or velocity, and larger cells are used in more uniform regions^24^. The mesh areas selected in this study align with the principles like, the smaller cell sizes (< 10,000 m^2^) improve resolution in zones with complex hydraulic features (e.g., urban areas, channels), while the larger cells (up to 19,778.33 m^2^) effectively model broader floodplain regions with lower spatial variability. The average cell area (10,197.35 m^2^) strikes a balance between computational efficiency and numerical accuracy.

### Time step selection

The model demonstrates adequate accuracy and stability with Courant numbers reaching up to 3.0 for the complete momentum equations and up to 5.0 when applying to the diffusion wave equations (HEC-RAS 2016). Under unsteady flow conditions, it is advisable to start with a coarser time step (ranging from 1 to 5 min) to ensure a balance between model stability and output accuracy. For modeling small urban areas, a Courant number of less than 1.0 is recommended to enhance output accuracy^24^. In this present study, a fixed time step of 1 min is utilized for the HEC-RAS model simulation to develop a stable and accurate model. A 1-minute time step effectively maintains a Courant number well within this recommended range, reducing numerical diffusion and ensuring the capture of dynamic flow characteristics. This choice balances computational efficiency with model accuracy, making it suitable for the spatial and temporal resolution required in the flood risk assessment of the study area.

### Manning’s roughness co-efficient (*n*)

In HEC-RAS 2D modeling, Manning’s *n* values represent the Roughness Coefficient for 2D flow areas that are typically linked to various land cover types. Several factors such as type and texture of the overland surface, the presence of pervious or impervious areas, and the depth of the 2D flow influences the Manning’s *n* values in 2D modeling. In this study, a Land Cover layer is created using the RAS Mapper tools, which can subsequently be utilized to establish roughness values for 2D flow areas. The Land Cover versus roughness table is formulated within this Land Cover layer. To create the Manning’s *n* versus Land Cover table, firstly navigate to the RAS Mapper, right-click on the desired Land Cover layer under Map Layers, and select Edit Land Cover Data (HEC-RAS 2D User Manual). This action will launch the Data Table Editor, where the Manning’s *n* value corresponding to each land cover type can be entered, as illustrated in Table 5.

US Geological survey prepared a technical manual for the Geospatial Stream Flow Model (GeoSFM) in which the roughness values are classified based upon the land cover type as shown in below table.

**Table 5:** Manning’s roughness values used for various land cover. (Source: GeoSFM manual, USGS).

Land Cover type	Manning’s Roughness (*n*)
Urban and Built-Up land	0.03
Dryland Cropland and Pasture	0.03
Irrigated Cropland and Pasture	0.035
Mixed Dryland/Irrigated Cropland and Pasture	0.033
Cropland/Grassland Mosaic	0.035
Cropland/woodland Mosaic	0.04
Grasssland	0.05
Shrubland	0.05
Mixed Shurbland/Grassland	0.05
Savanna	0.06
Deciduous Broadleaf Forest	0.1
Deciduous Needleleaf Forest	0.1
Evergreen Broadleaf Forest	0.12
Evergreen Needleleaf Forest	0.12
Mixed Forest	0.1
Water Bodies	0.035
Herbaceous Wetland	0.05
Wooded Wetland	0.05
Barren or Sparsely Vegetated	0.03
Tundra, Snow or Ice	0.05

### Python code

In the current study, XG Boost software library is used to predict the futuristic flood levels. For implementing this algorithm, different packages such as numpy, pandas have been used in the python code. By applying the XG Boost algorithm to the collected data, the prediction model is formed.

### Meteorological data

Moreover, Rainfall data are also collected which refers the information about the amount of precipitation that falls over a specific area during a certain period of time, usually measured in millimetres or inches. By analysing rainfall data, scientists and meteorologists can track patterns and trends in rainfall, predict droughts or floods, and make decisions to help communities to prepare themselves early and respond to changing weather conditions. The recurrence interval and intensity of flash flood events are mostly due to atmospheric and hydrological characteristics which remain elusive^32^.

The data given in Table 6 of temperature, relative humidity, specific humidity, wind, rainfall in inches, are from 1970 to 2020 for the location Narsingi, Gandipet, Hyderabad. The incorporation of 50 years of meteorological data enhances the robustness and reliability of flood prediction by capturing long-term climatic variability. This Data is uploaded in Google colab to get predicted flood levels for next 10 years. The Metrological data incorporated in the table describes the range (Minimum-Maximum) of individual parameter for each year from 1970 to 2020.

**Table 6:** Range of meteorological data of musi river from Gandipet Guage station.

Year	Temperature (in ^0^C) (Min–Max)	Relative humidity (in %) (Min–Max)	Specific humidity (in g/kg) (Min–Max)	Wind (in m/s) (Min–Max)	Rainfall (in mm) (Min–Max)
1970	21.53–33.19	41.21–88.46	6.69–21.79	2.90–5.81	0.002–10.32
1971	20.66–31.37	42.45–87.49	7.70-21.02	3.24–5.80	0.004–8.63
1972	21.45–32.65	42.56–81.27	6.98–20.20	3.05–6.12	0.06–7.73
1973	21.23–33.33	40.77–87.25	7.76–21.25	3.04–5.59	0.14–10.60
1974	20.41-32.388	40.17–82.79	6.51–20.83	2.87–5.45	0.04–8.55
1975	20.11–32.65	42.85–87.95	7.05–21.67	3.46–5.43	0.004–10.37
1976	20.93–31.94	42.12–87.66	7.37–21.10	2.80–5.17	0.02–9.99
1977	21.28–31.98	40.51–87.72	7.33–22.52	2.71–4.95	0.12–10.80
1978	20.54–31.82	42.98–86.61	6.84–21.79	2.96–5.20	0.23–9.96
1979	21.71–32.97	42.54–85.01	8.12–21.58	2.94–5.49	0.12–7.47
1980	21.28–33.73	43.66–86.06	7.51–22.23	2.96–5.30	0.12–9.33
1981	20.79–32.94	42.82–87.25	7.74–22.40	3.10–5.23	0.22–10.17
1982	21.65–32.38	46.13–87.89	8.08–20.92	2.44–5.20	0.07–7.27
1983	21.00-32.86	46.09–89.39	7.81–22.27	3.26–4.99	0.07-10.00
1984	21.16–33.61	43.22–86.81	7.94–21.57	3.33–5.57	0.05–9.16
1985	21.62–33.02	41.97–85.87	7.83–21.37	3.10–5.71	0.09–9.15
1986	21.26–32.36	44.84–83.89	8.16–20.96	3.09–5.31	0.14–8.81
1987	21.71–32.86	45.26–84.42	8.43–21.69	2.76–5.54	0.29–8.28
1988	22.54–33.58	44.78–88.40	7.74–22.44	3.18–5.09	0.05–11.80
1989	21.00-32.89	39.80-83.31	6.80-21.48	2.84–5.08	0.07–9.12
1990	22.08–32.87	41.64–87.95	7.19–21.76	3.18–5.58	0.04–10.16
1991	20.75–33.51	44.37–85.89	7.75–21.53	2.91–5.68	0.18–9.22
1992	20.94–32.78	40.33–88.05	7.03–21.54	2.84–4.89	0.02–9.36
1993	21.0-33.14	44.11–82.61	6.99–21.35	2.71–5.33	0.07–9.32
1994	21.02–32.96	42.53–88.91	6.85–21.93	2.85–5.40	0.009–11.56
1995	19.81–33.12	42.16–88.24	7.21–21.81	3.00-4.95	0.16–9.76
1996	21.17–32.50	42.88–85.16	7.41–21.02	3.05–4.96	0.04–9.38
1997	20.59–32.02	40.34–85.01	6.75–21.95	2.47–5.11	0.08–8.98
1998	21.61–33.61	42.64–85.95	7.64–22.27	3.04–4.96	0.004–8.94
1999	21.44–32.44	40.17–85.89	7.18–21.41	3.08–5.17	0.01–6.96
2000	21.48–32.52	42.42–84.10	7.15–20.80	2.48–4.70	0.03–9.17
2001	21.31–32.61	42.19–84.65	7.22–21.19	2.81–4.94	0.04–9.36
2002	21.20-32.91	43.23–84.91	7.73–21.07	2.38–5.18	0.06–8.40
2003	21.79–32.11	44.13–87.79	7.35–21.92	2.88–4.85	0.09–10.54
2004	21.53–32.76	40.04–88.15	7.37–21.73	2.90–4.74	0.010-9.00
2005	21.15–32.58	44.23–86.62	8.04–22.38	2.60–4.53	0.08–11.06
2006	22.36–33.06	40.96–89.40	7.04–22.07	2.60–5.04	0.01–10.52
2007	21.54–32.19	41.59–86.23	6.82–21.90	2.80–4.83	0.02–8.47
2008	21.09–32.50	45.69–87.45	7.52–22.01	2.51–4.81	0.07–8.82
2009	22.90-32.94	43.48–86.80	8.08–22.16	2.69–5.33	0.06–9.17
2010	22.05–33.47	44.50-88.79	7.68–22.59	2.77–4.69	0.18–9.91
2011	20.97–32.74	43.05–88.70	6.89–22.20	2.20–4.82	0.02–9.97
2012	20.57–33.18	40.77–85.25	7.60-21.99	2.66–5.21	0.07–8.92
2013	21.26–33.64	43.55–85.91	7.10-21.96	3.15–5.05	0.04–10.92
2014	21.15–33.77	46.05–83.70	7.84–21.57	3.04–5.03	0.21–9.06
2015	21.23–33.13	45.26–85.52	7.83–21.68	2.27–4.90	0.09–8.20
2016	21.55–32.90	41.39–86.44	7.03–22.37	2.65–4.60	0.04–10.65
2017	21.71–32.92	40.17–85.79	7.42–22.58	2.57–4.68	0.03–10.11
2018	22.41–32.70	40.17–84.32	6.75–22.42	2.66–5.16	0.01–9.39
2019	21.56–33.13	45.59–86.90	7.51–22.66	2.34–4.88	0.23–10.15
2020	21.21–32.19	46.24–87.69	8.31–22.37	2.73–4.64	0.09–11.42

### Flood susceptibility map

#### OpenTopography

OpenTopography is a web-based platform which allows user to access high resolution topography data to advance the understanding of Earth’s surface, vegetation and built environment. This web-based platform consists of regional and global (10–90 m resolution) datasets that are open and free for public access. In this study OpenTopography platform is used to extract the high-resolution topographic database with different raster visualizations like slope and Elevation through Shuttle Radar Topography Mission (SRTM GL1) Global 30 m. Arc-GIS is further used to visualize the above data.

#### ESRI 2020 land cover

A new 2020 Global Land Cover Map is created through a collaboration between Esri, Impact Observatory and Microsoft, utilizes high-resolution European Space Agency (ESA) Sentinel-2 satellite imagery and advanced machine learning techniques to provide a consistent, globally comprehensive view of land cover. In the present study the LULC map layers are downloaded from ESRI 2020 Land cover to analyse its influence on Flood susceptibility in the Osmansagar basin through Arc-GIS.

#### Uncertainty in land use

A signature refers to the unique fingerprint or pattern created by reflected wavelengths, which helps differentiate between various land cover types. These signatures serve as coordinates for pixels within an n-dimensional space, typically exhibiting a non-uniform arrangement that clusters into distinct regions. These signatures are used to demarcate the regions of that space occupied by each class of interest^6^.

When it comes to hydrological modeling, the uncertainty associated with land use data is influenced by how sensitive the model outputs are to changes in land use inputs. To evaluate the effect of this uncertainty, a sensitivity analysis using Monte Carlo simulation can be employed, assessing its impact on the hydrologic model^9^.

#### Climatic research unit (CRU) data

CRU includes high resolution gridded datasets of climate variables like precipitation, maximum and minimum temperature and cloud cover. This Unit consists of both pure and applied research, sponsored by external contracts and grants from academic funding councils, government departments, intergovernmental agencies, non-governmental organisations, and industries. Occurrence of flash floods is mostly due to excessive rainfall which leads to sudden rise in water level, so in the present study the precipitation datasets for the year 2020 have downloaded from CRU data to analyse the effect of rainfall on flood.

#### Flood vulnerability index (FVI)

In the calculation of exposure, Discharge, water level and Rainfall data is calculated from Gandipet Guage station. Drainage density, slope and elevation data is collected from raster grid maps of Osmansagar basin at random downstream points.

For susceptibility, the population density and literacy rate are collected from Telangana state statistical Abstract 2021 for different districts in Telangana. Number of buildings prone to flood is calculated using QGIS by intersection tool with input layer as clipped flood inundation map in shapefile format, with the building shapefile layer as overlay layer.

For Resilience, number of AWS situated district wise in Telangana is collected from a technical report submitted by Saha et al.^27^, entitled “Development of Real-time Rainfall Quality Monitoring for the Highly Dense AWS Network over Telangana”. Number of dams located district wise in Telangana is collected from a study done by Shalini entitled “Dams in Telangana State”.

## Results and discussions

### Flood inundation assessment

#### HEC-RAS

In the present study, for developing a hydraulic model to predict the effect of flood at downstream sections, HEC-RAS software is used for two-dimensional unsteady flow simulation by taking real time data of discharge at upstream section and friction slope at downstream section. After simulation, different output parameters such as variations in depth of flow, velocity gradient and water surface elevation in the downstream is obtained from the HEC RAS software.

**Fig. 9:**
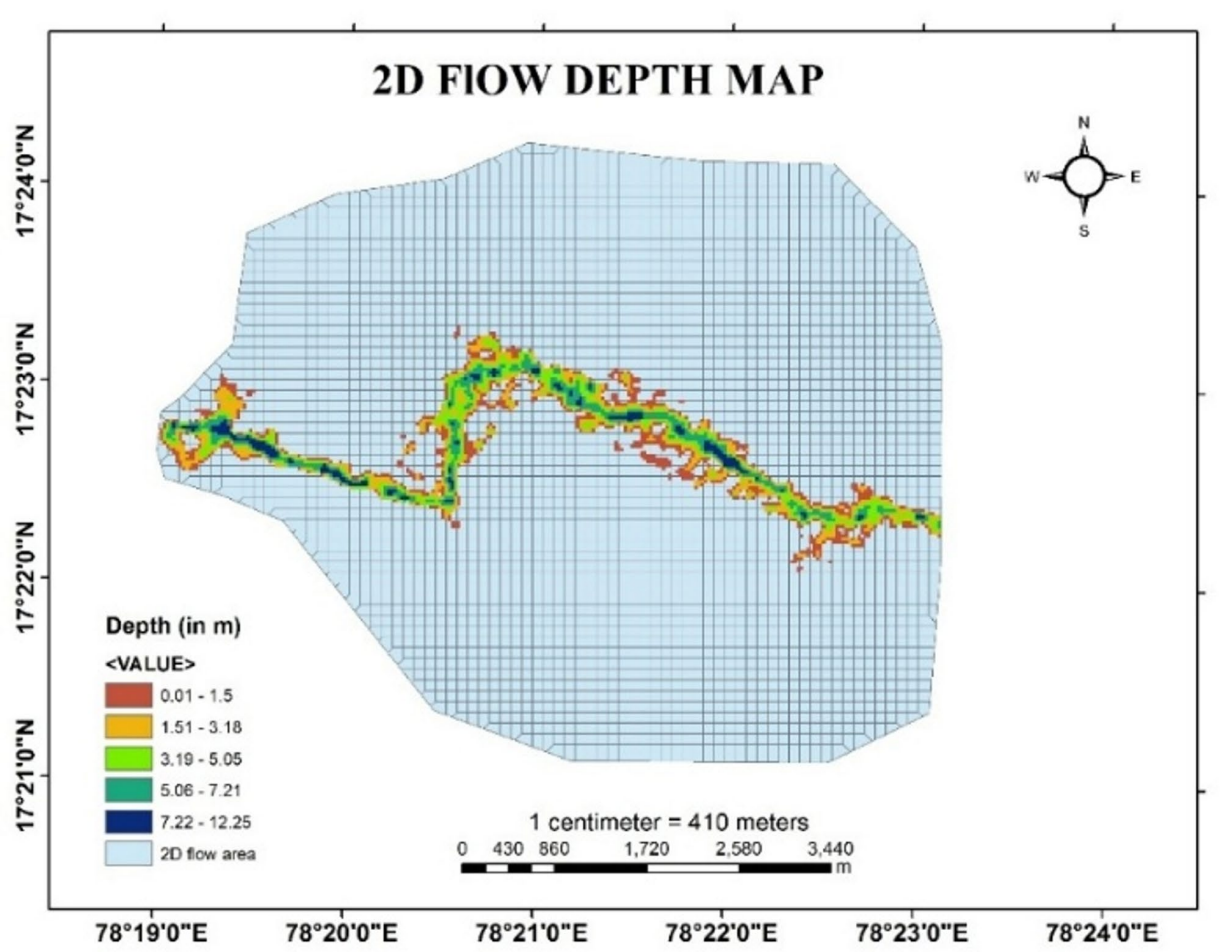
Flood Depth map of Musi River from Arc-Map software (version 10.3.1; Available at: https://www.esri.com/en-us/arcgis/products/arcgis-desktop/overview).

The flow profile at the downstream areas after the simulation in HEC-RAS for given discharge values is demonstrated in the Fig. 9. Variations in Flood depth, Flood velocity and water surface elevation in the map is indicated at every particular point of stream using colour indicator in RAS Mapper. The colour gradient in the stream describes the magnitude of depth, velocity and water surface elevation with respect to the scale given in the bottom right in the figures given below.

Initially the flow remained inside the bank, but eventually the flow undergoes to the bank full condition due to high discharge and later the stream rises to overbank flow conditions. Figure 9 shows the depth of flow varying up to 12.25 m depth which is continuously spreading over the low-lying areas for the given discharge and finally inundate the areas.

**Fig. 10:**
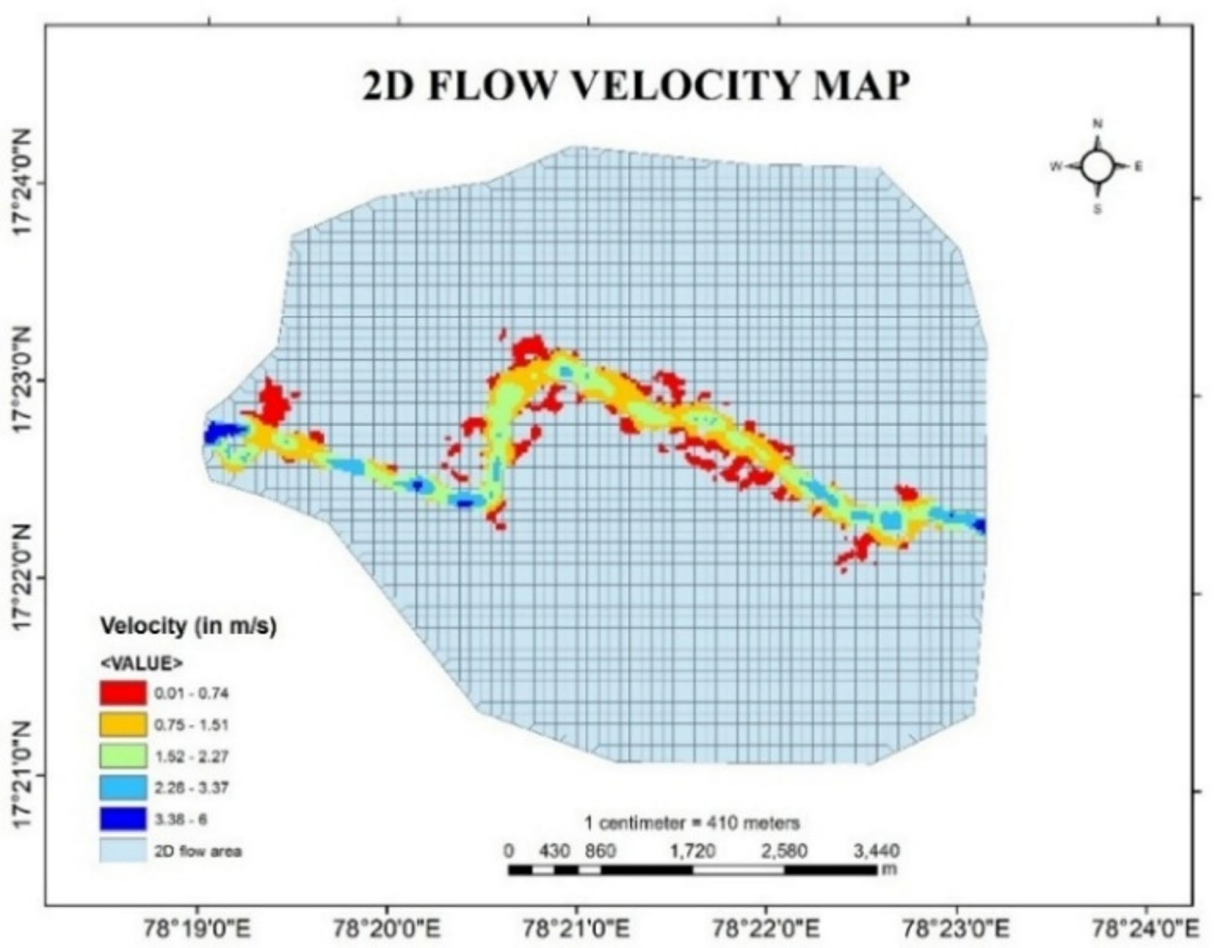
Flood Velocity map of Musi River from Arc-Map software (version 10.3.1; Available at: https://www.esri.com/en-us/arcgis/products/arcgis-desktop/overview).

From Fig. 10, it is observed that the stream velocity is high at the initial stages and decreasing less than 1 m/s in the downstream boundary. Due to this decrease in velocity from the upstream to downstream the flow becomes stagnant and leads to inundation of the stream surrounding areas.

**Fig. 11:**
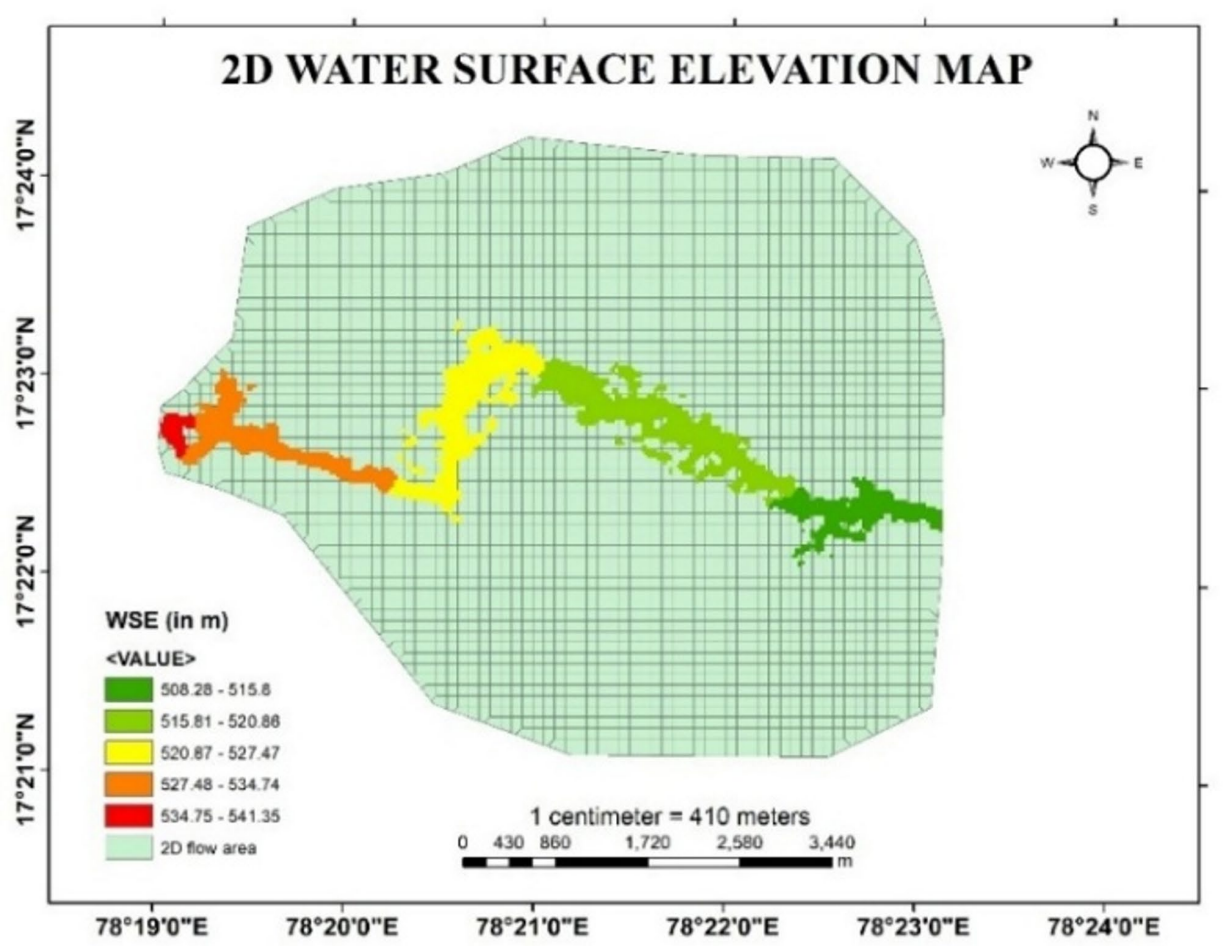
Water surface elevation map of Musi River from Arc-Map software (version 10.3.1; Available at: https://www.esri.com/en-us/arcgis/products/arcgis-desktop/overview).

From the Fig. 11, it is noticed that the water surface elevation throughout the stream is varying from peak level and decreasing gradually. The magnitude of WSE is varying from 508.28 m to 541.35 m, which indicates a decreasing trend in WSE from upstream to the downstream section. The relatively lower WSE observed in the downstream sections attributed to flow spreading under overbank flow conditions, which causes the flow to disperse laterally and reduce WSE.  .

The above flow profiles indicate the effected low-lying areas after the flood produced by the discharge at the upstream section. So, the people from this flood prone areas needs to take flood mitigation measures to survive against the hazardous flood situations. From the study, it has been observed that there are huge chances of risk to the buildings and houses in the flood prone areas that affects as per the flow profile. So, to avoid those drastic situations, protection measures have to be initiated to safeguard the buildings from floods.

#### Validation of model

To validate the simulated flood levels in the downstream, predicted depth of water from HEC-RAS and observed water level data from gauge station is compared. The statistical analysis resulted in a Mean absolute deviation (MAD) of 7.13 m and Root mean square error (RMSE) of 7.14 m. The close agreement between MAD and RMSE indicates a uniform distribution of errors without significant outliers. This reflects the model’s capability to reasonably simulate the temporal variation of flood levels during the event. Table 7 shows the validation of HEC-RAS simulated flow depth against observed water level data.

**Table 7:** Validation of 2D HEC-RAS model.

Time (in hrs)	Simulated flow Depth (in m)	Observed flow depth (in m)
2	9.14	16.84
4	9.335	16.84
6	9.39	16.84
8	9.5	16.84
10	9.652	16.84
12	10.2	16.82
14	10.631	16.85
16	10.54	16.93
18	10.03	16.93
20	9.6	16.92
22	9.4	16.92

The RMSE value of 7.13 m is relatively high, but the primary objective of this study is to assess the flood inundation extent rather than precise depth prediction. For such spatial analysis, 2D hydrodynamic modeling is more suitable as it effectively captures lateral flood propagation^19^. In contrast, 1D models are generally preferred for vertical or point-based flood depth analysis. Therefore, the chosen 2D approach aligns well with the study’s focus on inundation mapping over complex terrain.

#### QGIS

After downloading the shape file layer of study area, building shape layer is added in to the QGIS workspace. Road shapefile layer is also added for reference purpose. Then a mesh layer has been added to the workspace to overlay the inundation map extracted from HEC-RAS to determine the regions inundated due to flood.

**Fig. 12:**
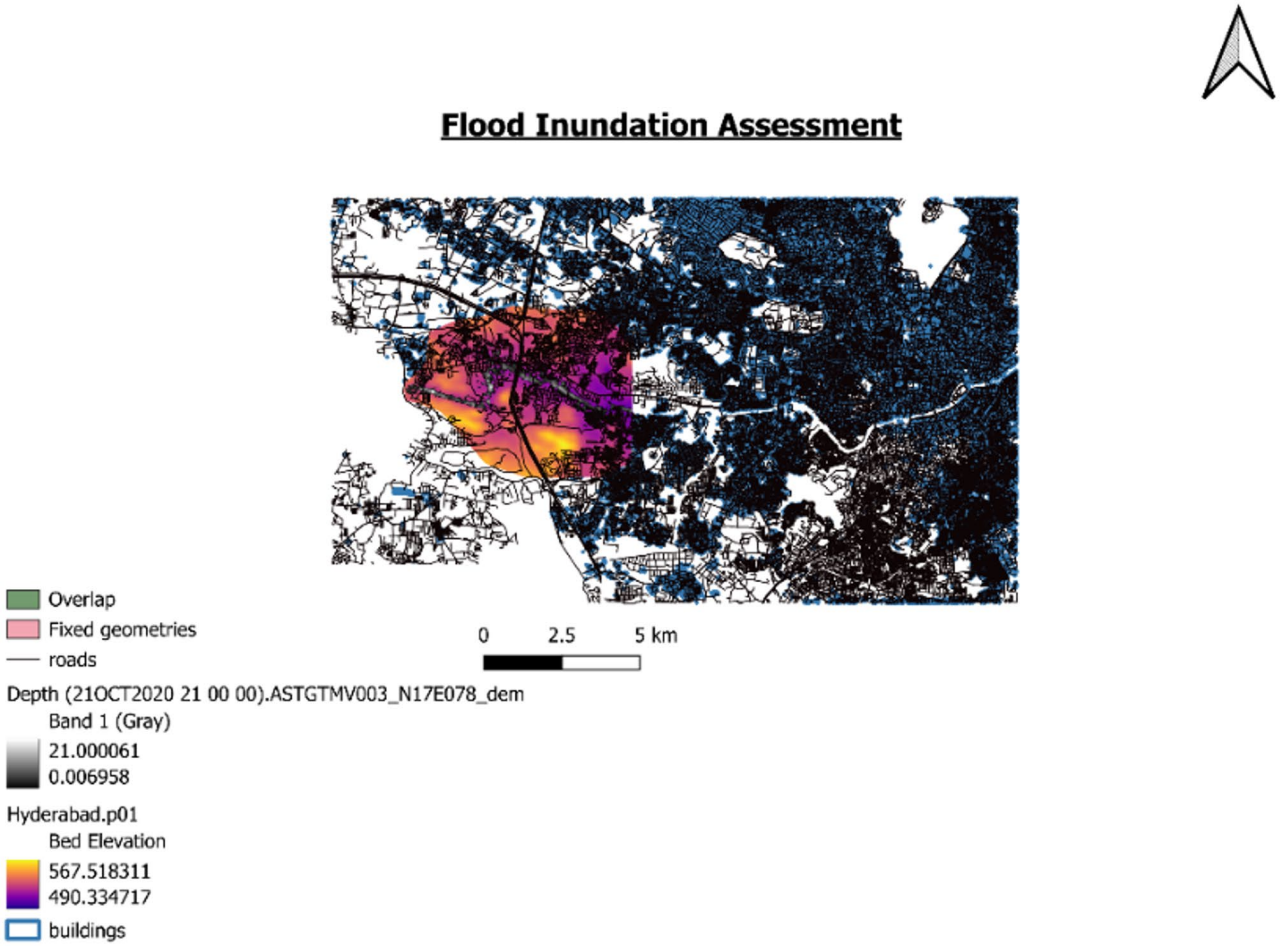
Inundation Map through overlay analysis from QGIS software (version 3.34.1; Available at: https://qgis.org/download/).

Figure 12 shows the bed elevation of the inundation map overlayed with building shapefile layer. The violet colour represents more depth and orange represents least depth of submergence level. The areas under violet colour in the Fig. 12 are flood prone areas. So, the people under the violet region should take flood mitigation measures.

The spatial overlay of the HEC-RAS-derived inundation map with the building footprint shapefile in QGIS identified that, 104 buildings as exposed to flood risk within the study area for the discharge of 1652.29 m^3^/s. These buildings intersect with the simulated flood extent, indicating potential structural and functional vulnerability during flood events.

#### XG boost

**Fig. 13:**
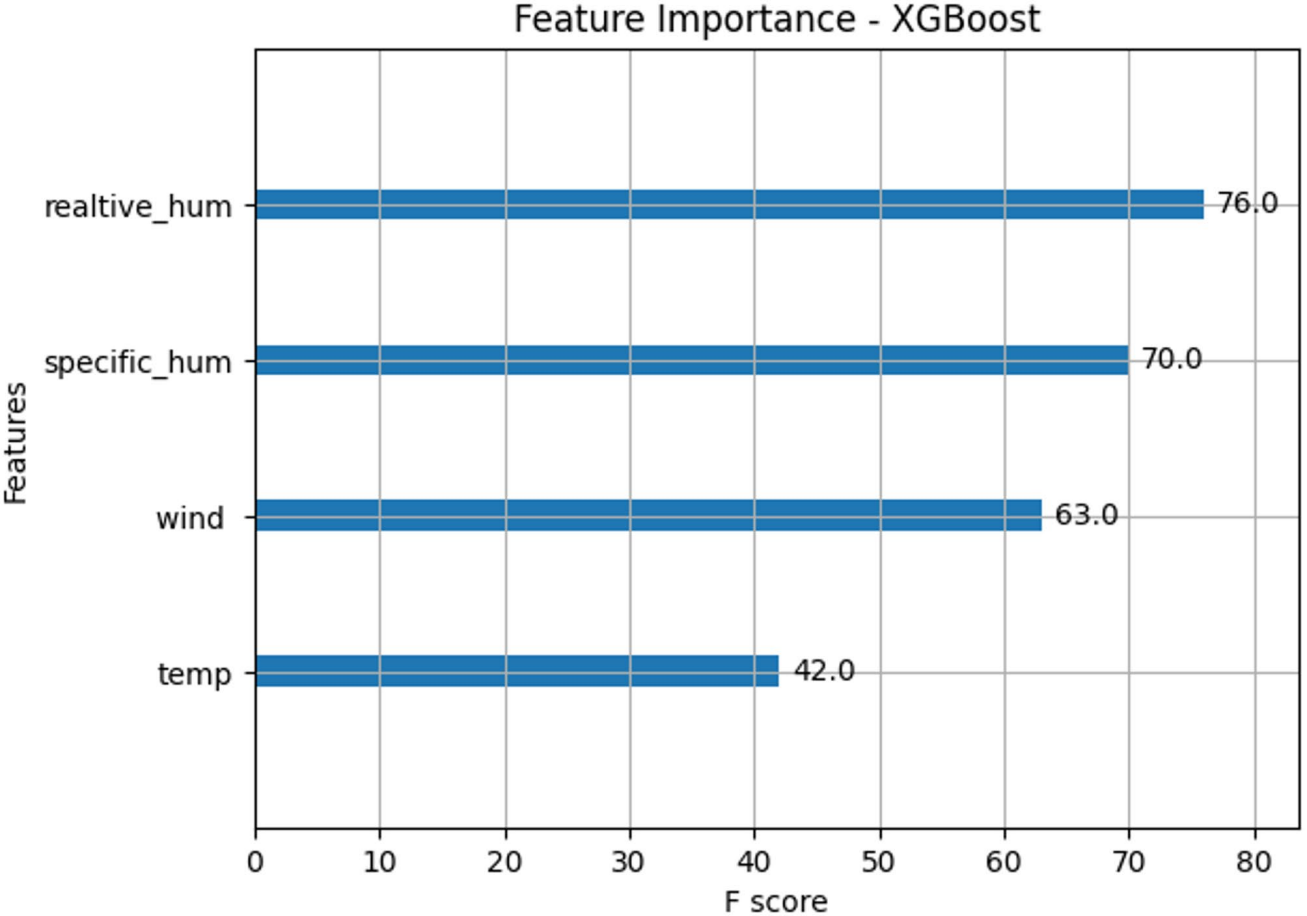
Feature importance Visualization in XGBoost.

In the present study, the XGBoost software library is used with past rainfall data from the year 1970 to 2020 as input in the python code. By importing pandas library and numerical python (numpy) library, the python code has been initiated Firstly, in XG Boost software, python code is written by including Temperature, Relative Humidity, Specific Humidity and Wind as input parameters from 1970 to 2020 to observe the effect of flash floods during the year 2020 to 2030. This python code has successfully executed and ready to predict the flood levels. This prediction model is further verified by forecasting the flood levels during the years 2020 to 2030. Figure 13 demonstrates the importance of features in construction of boosted decision tree in XGBoost.

**Fig. 14:**
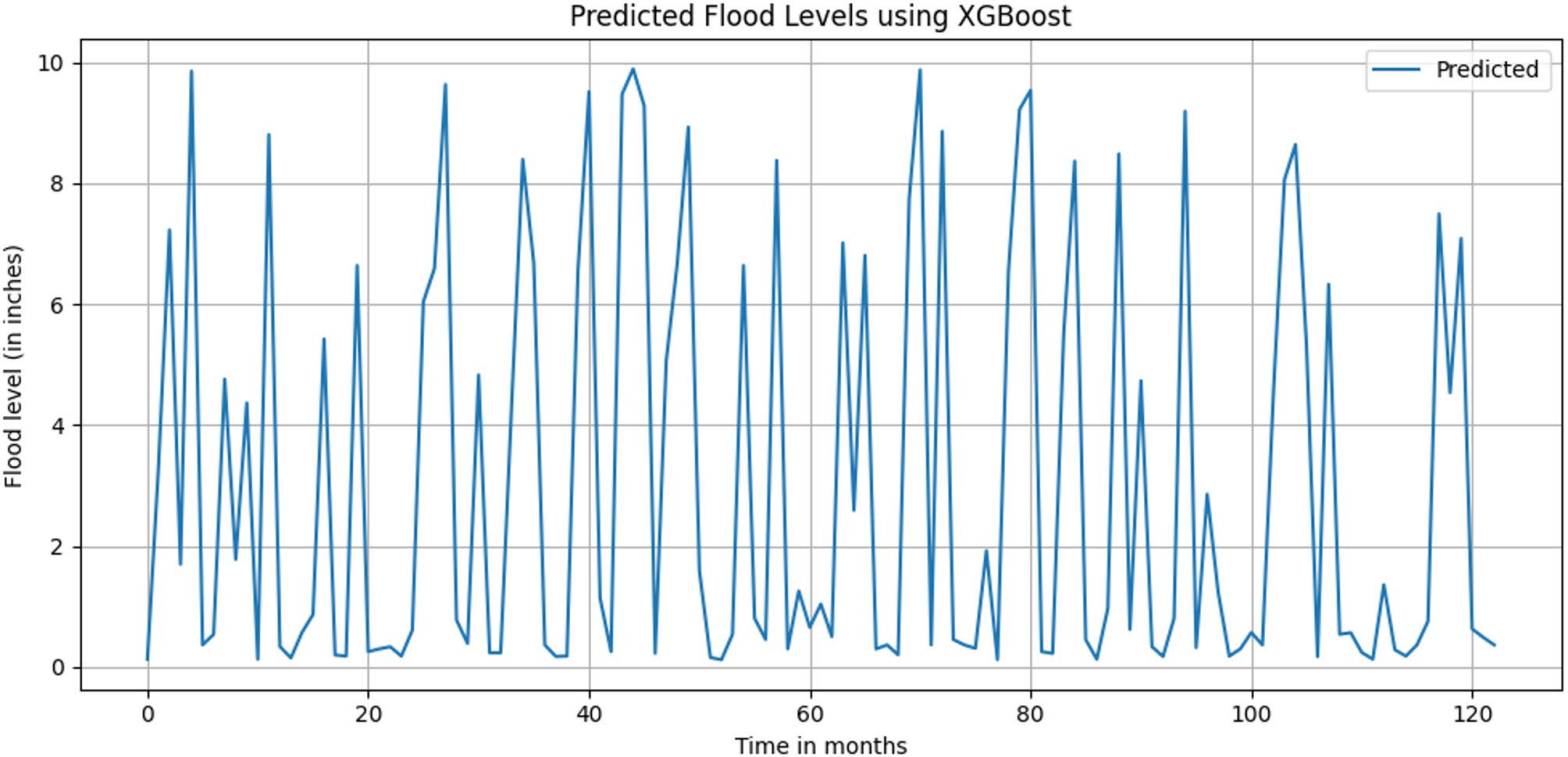
Predicted Flood levels at Narsingi and Gandipet areas (After 2020).

Figure 14 describes the predicted flood levels in inches during the time period. In Fig. 14, the prediction of Flood levels for 10 years (120 months) is on X Axis, with respect to predicted Flood Level in inches is on Y Axis. In the graph it is clearly visible that the flood level is continuously fluctuating with respect to the given time period.

The performance of the XGBoost regression model is then evaluated using standard error metrics. The model yielded an R^2^ score of 0.945, indicating a strong correlation between the predicted and actual flood level values. The RMSE is found to be 0.795 mm, while the Mean Absolute Error (MAE) is 0.514 mm. These low error values suggest that the model is capable of accurately capturing the non-linear relationships among meteorological variables such as temperature, humidity, specific humidity, and wind speed.

#### Flood susceptibility map

The Osmansagar basin partially situated in the district of Hyderabad and partially situated in the Ranga Reddy district. In the present study five influencing factors like Elevation, slope, land use and land cover, Annual rainfall and Drainage density are considered to determine the flood susceptibility of Osmansagar basin. Thematic maps of influencing factors are extracted using ARC-GIS and reclassified individually based upon intensity of risk levels from 1 to 5 in Arc-GIS as shown in Table 8. These processed maps, derived from various geographical and hydrological features, serve as essential flood hazard indicators to understand and visualize flood risk in the study area^14^.

**Table 8:** Classification of flood risk level from 1–5.

Intensity of Risk level	Meaning
1	Very low chances of flood risk
2	Low chances of flood risk
3	Moderate chances of flood risk
4	High chances of flood risk
5	Very high chances of flood risk

#### Slope and elevation

**Fig. 15:**
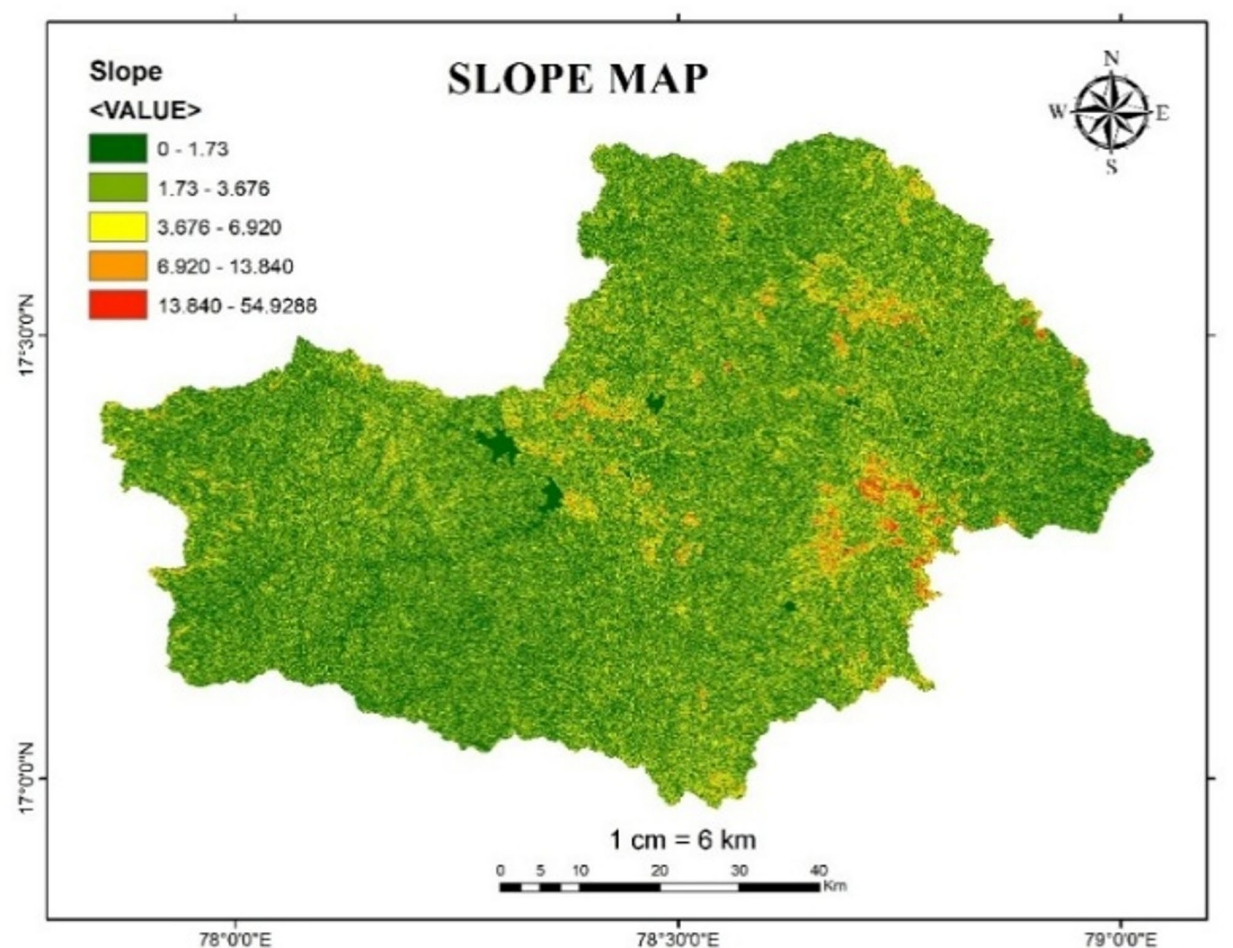
Slope map of Osmansagar Basin from Arc-Map software (version 10.3.1; Available at: https://www.esri.com/en-us/arcgis/products/arcgis-desktop/overview).

**Fig. 16:**
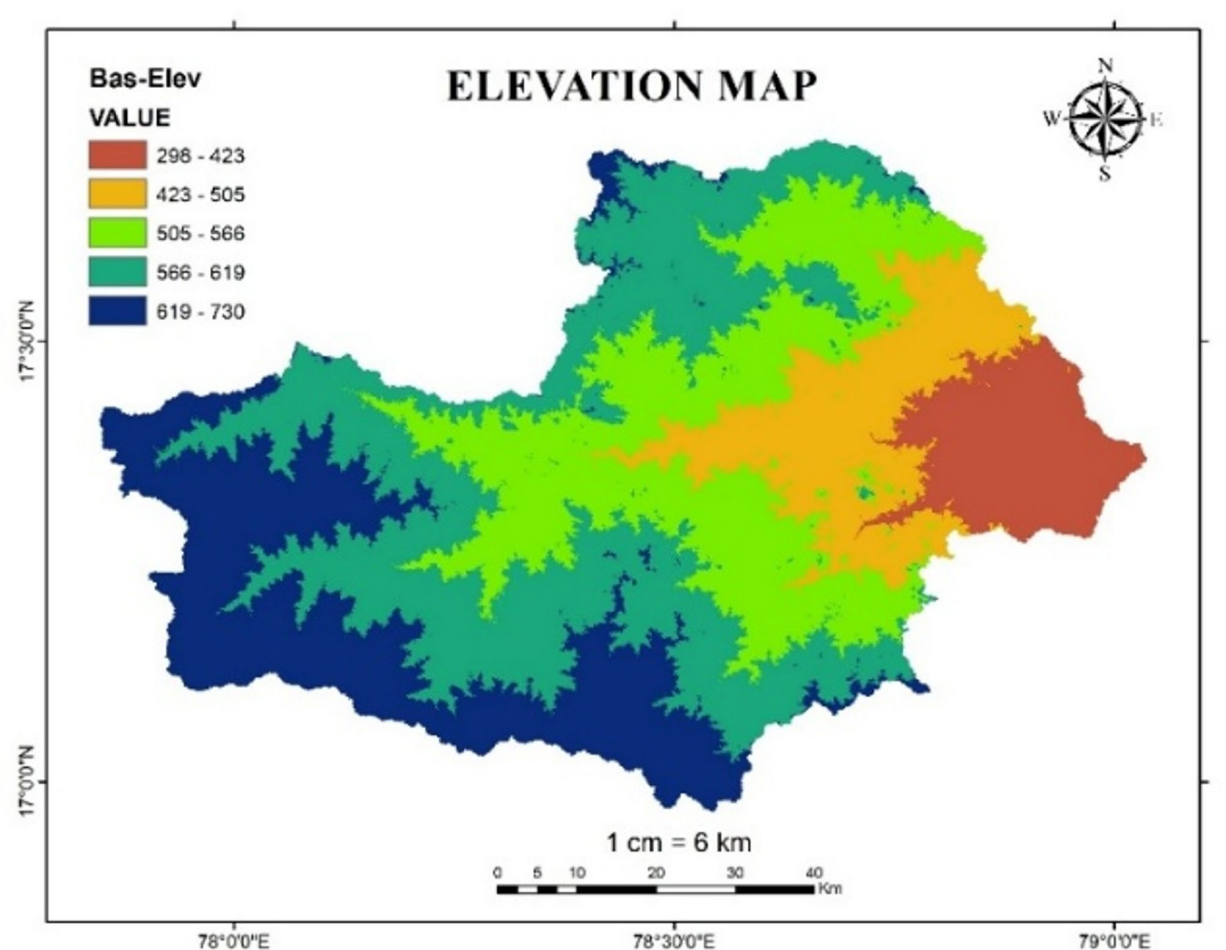
Elevation map of Osmansagar Basin from Arc-Map software (version 10.3.1; Available at: https://www.esri.com/en-us/arcgis/products/arcgis-desktop/overview).

Slope and elevation are the critical factors in occurrence of flood. Higher elevation and slope affect the direction and depth of water movement, leading to increased runoff, while lower elevation slope can result in waterlogging^30^. In this study Slope map and Elevation map of Osmansagar basin is reclassified based upon flood risk level as shown in the Tables 9 and 10. Figures 15 and 16 shows the Slope map and Elevation map of Osmansagar basin.

**Table 9:** Flood risk level based on slope ratio.

Slope value	Flood risk level
0-1.73	5
1.73–3.676	4
3.676–6.920	3
6.920–13.840	2
13.840-54.9288	1

**Table 10:** Flood risk level based on elevation factor.

Elevation value (in m)	Flood risk level
298–423	5
423–505	4
505–566	3
566 − 519	2
619–730	1

#### Land use and land cover

In flood hazard mapping, land use and land cover map plays a key role because a land use map can assist to recognise the areas that are vulnerable to flooding. Land-use patterns act as protective shields by reducing water retention time and potentially amplifying flood intensity^8^. The LULC map is generated using data from the year 2024, to represent the most current surface conditions of the study area. This real-time LULC data improves the spatial accuracy of the analysis by capturing the present-day conditions, enhancing the reliability and relevance of the flood hazard and risk mapping outcomes.

**Fig. 17:**
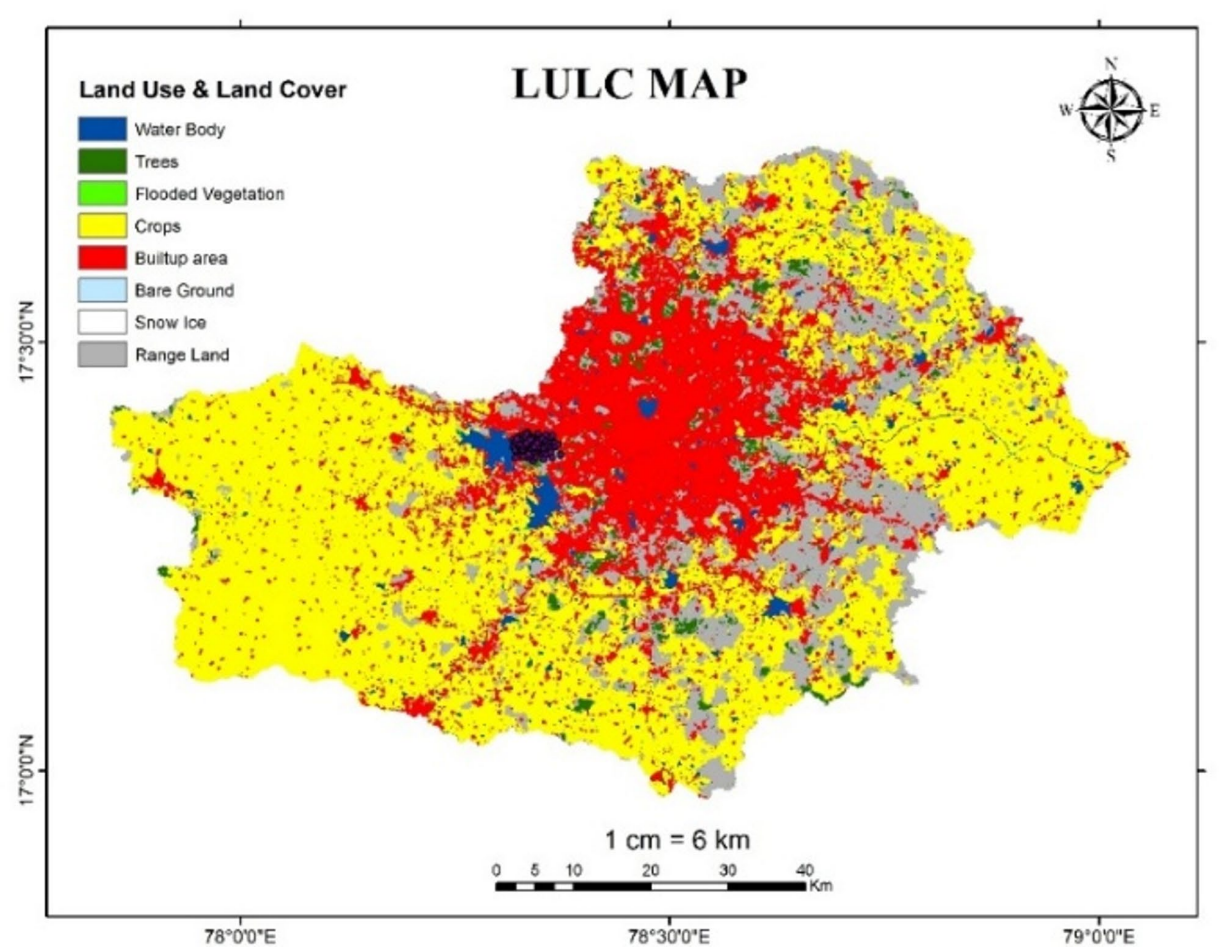
LULC map of Osmansagar Basin from Arc-Map software (version 10.3.1; Available at: https://www.esri.com/en-us/arcgis/products/arcgis-desktop/overview).

In the present study LULC map is reclassified with respect to flood risk level from 1 to 5 according to the usage of land as shown in Table 11. Figure 17 describes the land use and land cover in Osmansagar basin.

**Table 11:** Flood risk level based on land use type.

Land use type	Flood risk level
Waterbody	5
Bareland	4
Built up area	3
Crop land	2
Flooded Vegetation	1
Range land	0
Snow ice	0
Trees	0

#### Annual rainfall

Rainfall is the key source of flood occurrence. The intensity and duration of rainfall describes the possibility of flood. During the excessive rainfall, the natural drainage systems like rivers and streams leads to overbank flow condition when the ground exceeds its absorption capacity. The volume of runoff is directly proportional to the intensity of rainfall in an area. Due to accumulation of rainwater, water level rises in rivers and lakes that leads to a potential breach in banks and dams which initiates river-based flooding.

**Fig. 18:**
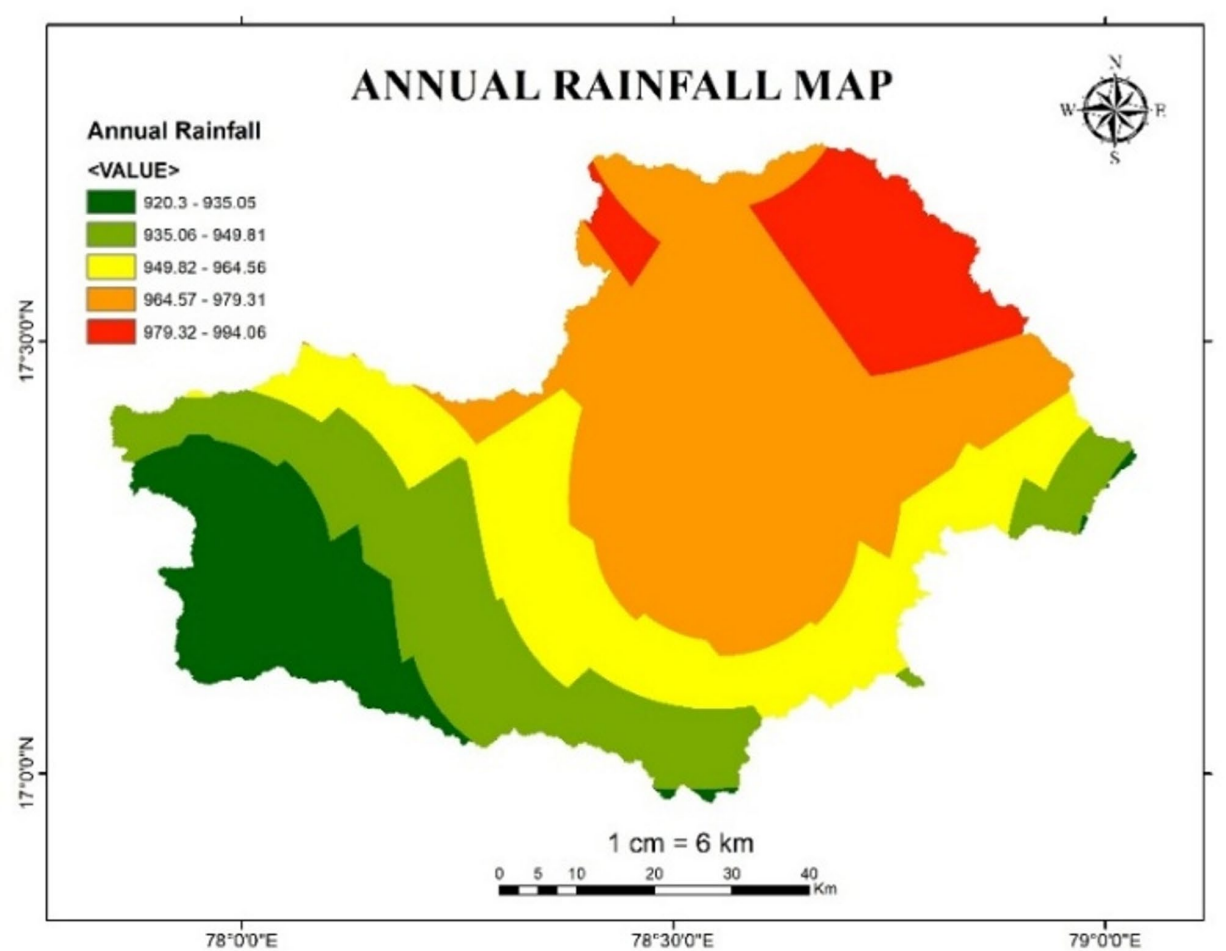
Annual Rainfall map of Osmansagar Basin of year 2020 from Arc-Map software (version 10.3.1; Available at: https://www.esri.com/en-us/arcgis/products/arcgis-desktop/overview).

To ensure the reliability of the flood risk assessment, recent and relevant datasets were utilized. The annual rainfall map is generated using data from the year 2020, when Hyderabad experienced its most extreme rainfall event in 117 years, with an average daily rainfall of 19.2 cm. Using this high-impact event as the basis for flood hazard mapping ensures that the resulting map reflects a realistic worst-case scenario. This provides a valuable reference for decision-makers to identify peak-risk zones and to develop effective mitigation and planning strategies under severe flood conditions. Figure 18 describes the Annual Rainfall map of year 2020 for Osmansagar basin. Table 12 shows the reclassified rainfall values based upon flood risk level.

**Table 12:** Flood risk level based on annual rainfall in mm.

Annual Rainfall (in mm)	Flood risk level
920.3 −935.05	1
935.06-949.81	2
949.82-964.56	3
964.57-979.31	4
979.32-994.06	5

#### Drainage density

**Fig. 19:**
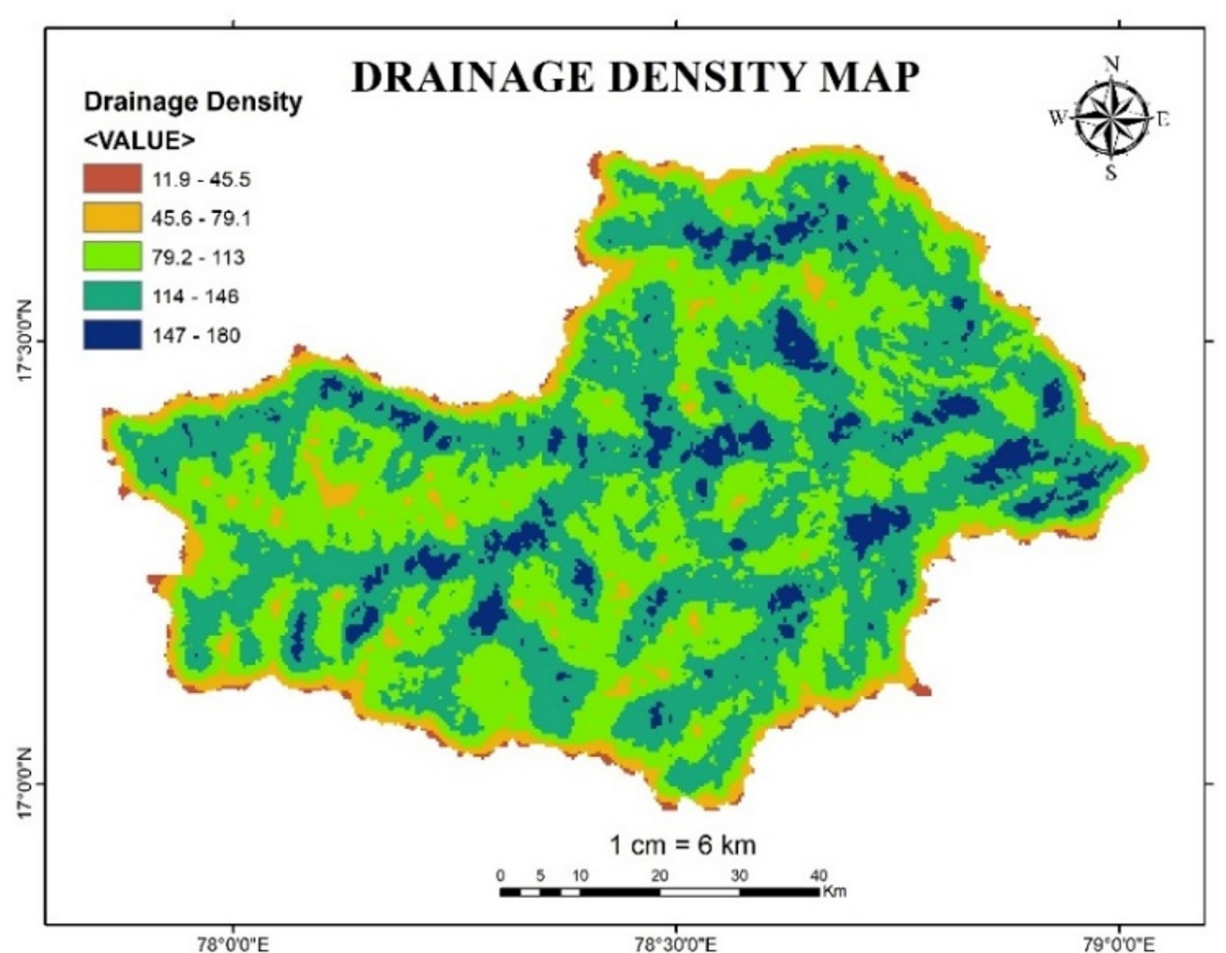
Drainage Density map of Osmansagar Basin from Arc-Map software (version 10.3.1; Available at: https://www.esri.com/en-us/arcgis/products/arcgis-desktop/overview).

Drainage density is also a key aspect which initiates the high flood scenarios. Higher drainage density leads to a high possibility of flood risk. During the peak rainfall in a particular basin water drains out from the basin more quickly due to the large network of smaller streams which leads to a potential flood. Figure 19 indicates the drainage density map of Osmansagar basin. Table 13 describes the reclassified values of Drainage density based upon the flood risk level.

**Table 13:** Flood risk level based on drainage Density.

Drainage Density (km/km^2^)	Flood risk level
11.9–45.5	1
45.6–79.1	2
79.2–113	3
114–146	4
147–180	5

#### Computation of weights

After reclassification of all maps, weights of each influencing parameter are determined based on expert opinion, subject knowledge, previous literatures and spatial features of study area etc. Table 14 shows the weights of influencing parameters on flood occurrence.

**Table 14:** Weights of influencing parameter on flood occurrence.

Variable Name	Weight
LULC	15
Drainage density	20
Slope	15
Rainfall	30
Elevation	20

**Fig. 20:**
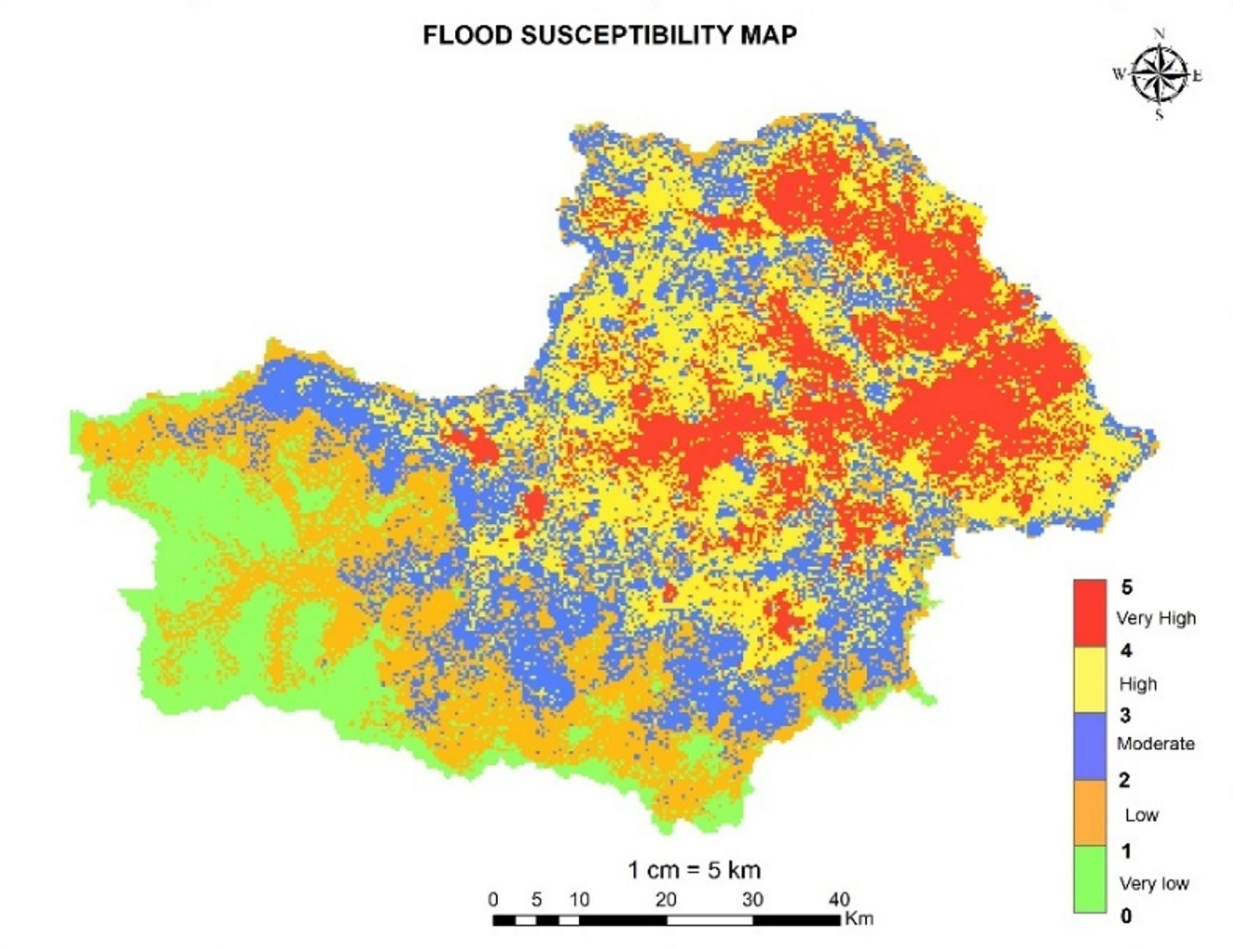
Flood Susceptibility Map of Osmansagar Basin from Arc-Map software (version 10.3.1; Available at: https://www.esri.com/en-us/arcgis/products/arcgis-desktop/overview).

The results illustrate that 1036.647 km^2^ area (17.77% of total study area) is prone to very high flood hazard, 1507.42 sq.km area comes under highly vulnerable zone to flood (25.85%), 1293.306 sq.km area comes under moderate vulnerable zone to flood (22.18%), 1210.76 sq.km area comes under low vulnerable zone to flood (20.76%) and remaining 741.98 km^2^ area comes under very low vulnerable flood hazard zone (12.72%). This indicates that more than 65% of the Osmansagar basin is vulnerable to flooding. Figure 20 shows the Flood susceptibility map of Osmansagar basin. The figure legend represents a colour coded flood susceptibility scale from 0 to 1 as very low flood risk zone, 1–2 as low flood risk zone, 3–4 as moderate flood risk zone, 3–4 as high flood risk zone and 4–5 as very high flood risk zone.

### Sensitivity analysis

The baseline weights are maintained for comparison with the modified weights of influencing parameters in the sensitivity analysis. Table 15 shows the percentage of weights considered during classification of flood risk zones. Table 16 shows the extent of flood with respect to risk zones for different weights of influencing parameters.

**Table 15:** Weights variations of influencing parameters (in %).

Scenario	Rainfall	LULC	Slope	Elevation	Drainage Density
Base	30	15	15	20	20
High rainfall	40	12.5	12.5	17.5	17.5
Low rainfall	20	17.5	17.5	22.5	22.5
High LULC	27.5	25	12.5	17.5	17.5
Low LULC	32.5	5	17.5	22.5	22.5
High slope	27.5	12.5	25	17.5	17.5
Low slope	32.5	17.5	5	22.5	22.5
High Elevation	27.5	12.5	12.5	30	17.5
Low elevation	32.5	17.5	17.5	10	22.5
High Drainage Density	27.5	12.5	12.5	17.5	30
Low Drainage density	32.5	17.5	17.5	22.5	10

**Table 16:** Flood extent for different flood risk zones at varying weights **(**in sq.km).

Zone	1	2	3	4	5
Base	741.98	1210.80	1293.3	1507.40	1036.60
High Rainfall	773.77	1118.37	1214.13	1619.98	1062.90
Low Rainfall	541.54	1206.05	1552.93	1609.49	879.41
High LULC	405.73	1395.80	1555.85	1522.59	908.98
Low LULC	721.94	1066.22	1433.65	1453.20	1114.53
High Slope	495.02	1280.93	1477.42	1483.44	1052.33
Low Slope	940.45	1224.91	1268.21	1343.36	1013.48
High Elevation	932.32	982.678	1297.98	1430.13	1147.08
Low Elevation	738.05	1144.71	1303.94	1410.39	1192.67
High Drainage density	649.76	1190.47	1402.72	1522.14	1024.88
Low Drainage density	820.37	1097.22	1261.63	1498.96	1111.60

**Fig. 21:**
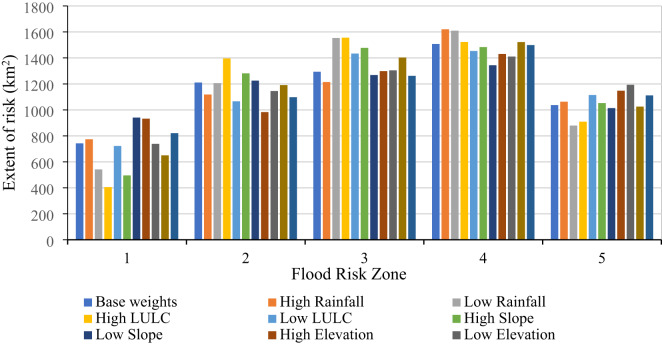
Sensitivity of flood extent to variations in weights of influencing factors.

The sensitivity plot illustrates the influence of various influencing parameters on the extent of risk across classified flood risk zones. From Fig. 21, it is clear that impact of low rainfall is significantly reduced in zone 1 and 2, heavy rainfall having an increasing extent of risk in zone 4. High LULC causes a noticeable increase in flood extent majorly in zone 2 and 3, low LULC having a decrease in flood extent that describes the risk of urbanization. Slope and elevation show moderate influence on flood extent. High Drainage density and low drainage density is also affecting the flood extent in zone 3 and 4 with less impact.

### Flood vulnerability index (FVI)

To calculate the vulnerability of each individual indicator, the indicator values are normalized from 0 to 1 based on its maximum and minimum indicator value. Basically, for FVI calculation, 11 indicators are selected in this study. Indicators like water level, Discharge, Drainage density, rainfall and population density are directly influencing the flood vulnerability. So, for normalization of directly influencing indicators below formula is used:

1 $$\:{\text{X}\:}_{\text{N}\text{o}\text{r}\text{m}\text{a}\text{l}\text{i}\text{z}\text{e}\text{d}}\:\frac{X-{X}_{Minimum}}{{X}_{Maximum}-{X}_{Minimum}}$$

For the indicators like Elevation, slope and literacy rate which are inversely affects the flood vulnerability is normalized using below formula:

2 $$\:{\text{X}\:}_{\text{N}\text{o}\text{r}\text{m}\text{a}\text{l}\text{i}\text{z}\text{e}\text{d}}\:\frac{{X}_{Maximum}-X}{{X}_{Maximum}-{X}_{Minimum}}$$

where, $\:X$ = Actual value of Indicator, $\:{X}_{Maximum}$ = Maximum value in the dataset of Indicator values, $\:{X}_{Minimum}$ = Minimum value in the dataset of Indicator values.

For the indicators considered for resilience, such as number of AWS and dams are normalized using equation (i), as the FVI formula consists a negative sign for Resilience.

3 $${\rm FVI} ={\rm Exposure}+{\rm Susceptibility}-{\rm Resilience}$$

FVI = 0.548327222 + 0.368- 0.356666667.

FVI = 0.5596.

This Index indicates the selected area for calculating FVI is moderate to highly vulnerable to the floods. This value indicates is the selected region is not most vulnerable but still it faces significant flood risk.

### Sensitivity analysis

To evaluate the influence of each indicator on the FVI, the indicators categorized under exposure, susceptibility, and resilience components, underwent normalization ranging from 0 to 1 using min-max scaling^22^. Using normalized values of indicators, the base FVI is calculated and then the FVI is recalculated iteratively by excluding one indicator at a time, and the resulting variation in FVI values were recorded as shown in the Table 17.

**Table 17:** Sensitivity analysis of FVI using leave-one-out-method.

Removed Indicator	Exposure Sum	Susceptibility Sum	Resilience Sum	FVI	Sensitivity
Base	0.54	0.36	0.35	0.55	-
Exp-Water Level	0.58	0.36	0.35	0.59	5.83
Exp-Discharge	0.57	0.36	0.35	0.58	5.34
Exp-Drainage Density	0.52	0.36	0.35	0.54	3.27
Exp-Rainfall	0.55	0.36	0.35	0.57	1.72
Exp-Elevation	0.53	0.36	0.35	0.54	2.40
Exp-Slope	0.50	0.36	0.35	0.52	7.22
Sus-Population Density	0.54	0.05	0.35	0.24	56.46
Sus-Literacy Rate	0.54	0.55	0.35	0.74	32.87
Sus-Infrastructure	0.54	0.5	0.35	0.69	23.58
Res-AWS	0.54	0.36	0.33	0.58	4.76
Res-Dams	0.54	0.36	0.38	0.53	4.76

**Fig. 22:**
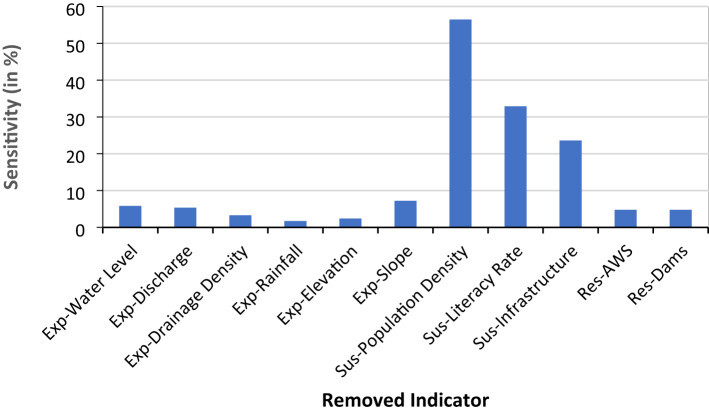
Sensitivity Analysis of FVI by removing individual indicators.

The results reveal that socio-economic indicators, particularly population density, literacy rate, and infrastructure, exhibit the highest sensitivity. These indicators, when excluded, caused significant changes in the overall FVI score for Hyderabad. This heightened sensitivity is attributed to the urban character of Hyderabad, where population density and infrastructure development are substantially higher than in other districts. Figure 22 shows the bar graph describing the sensitivity of FVI in response to removal of individual indicator.

On the contrary, indicators such as rainfall, discharge, slope, elevation, and drainage density having lower sensitivity. This is primarily because these factors exhibit less variation spatially when compared to socio-economic indicators. Additionally, in urban cities like Hyderabad, topographical and hydrological variations are often minimized due to extensive land development, engineered flood control systems, and reduced natural surface variation.

The sensitivity analysis thus highlights the dominant role of socio-economic vulnerability in determining flood risk in urban centers. These findings emphasize the importance of targeting densely populated and highly built-up zones for flood resilience planning.

### Correlation of FVI with actual flood damage indicators

The FVI developed in this study were further evaluated by correlating it with actual flood damage indicators such as the infrastructure damages due to high flood scenarios.

### High correlation with socio-economic damage

The sensitivity analysis of FVI revealed that indicators like population density, infrastructure density, and literacy rate showed the highest influence on the FVI score. Notably, the study area also experienced significant structural damages and displacement during past flood events, indicating a strong positive correlation between the FVI and actual damage.

### Low sensitivity of topographic indicators

In contrast, indicators like elevation, slope, rainfall, and drainage density exhibited lower sensitivity in FVI. This corresponds well with the minimal observed spatial variation in topographic and hydrological damage patterns across urban Hyderabad, where engineered structures have reduced natural variability. Therefore, their weaker correlation with actual flood damage aligns with their lesser influence in urban environments.

Rangari et al.^25^ developed an urban flood hazard assessment framework that integrated scale invariance theory with hydrodynamic modeling. The large-scale intensity-duration-frequency (IDF) relationships are derived using non-central moment estimation, from which scaling components were extracted to build scaled IDF models. These models were used to generate synthetic rainfall hyetographs, which served as an input to HECRAS 1D-2D simulations for urban flood modeling. The primary objective of their study is to develop a 1D-2D urban flood model to identify flood susceptible zones and evaluate the potential escalation of flood hazard by integrating GIS with rainfall-runoff model and hydraulic model HEC-RAS under climate change scenarios.

Flood hazard estimation in their study is based on inundation depth, flow velocity and combined effect processed using ArcGIS. The flood inundation depth and flood flow velocity maps which are processed in Arc-GIS combined both parameters to develop a flood-hazard classification for each flood condition.

In contrast present study adopts a data driven and hybrid modelling approach by combining hydrodynamic simulation and machine learning. Firstly, a hydrodynamic model is developed using discharge data through HEC-RAS 2D simulation. The flood depth map exported from HEC-RAS and used for overlay analysis with building shapefile layers in QGIS to assess the spatial extent of flooding and its impact on infrastructure.

Secondly, XGBoost, a robust machine learning algorithm is employed to forecast flood levels for 10-year period sing metrological variables. Additionally, a flood hazard map is generated using five influencing factors such as Annual rainfall, Elevation, slope, Land use and land cover (LULC) and Drainage density. This flood hazard map is reclassified in to five risk zones (very low to very high) and the area under each flood risk zone is computed. Furthermore, a FVI is calculated based on an equation. Various indicators are used to quantify exposure, susceptibility and resilience respectively, offering a more holistic view of flood vulnerability.

Thus, Rangari et al.^25^ method emphasizes a scale-based modelling framework with synthetic inputs to explore future flood risk under climate change scenarios, but the present study is a data driven framework combining machine learning, hydrodynamic modelling and GIS based spatial analysis. Both approaches offer valuable insights, where Rangari’s for scenario-based hazard estimation and generalized scaling behaviour and the present study for its practical application, spatial precision and integration of vulnerability indicators, together contributing to a more comprehensive understanding of urban flood risk.

**Fig. 23:**
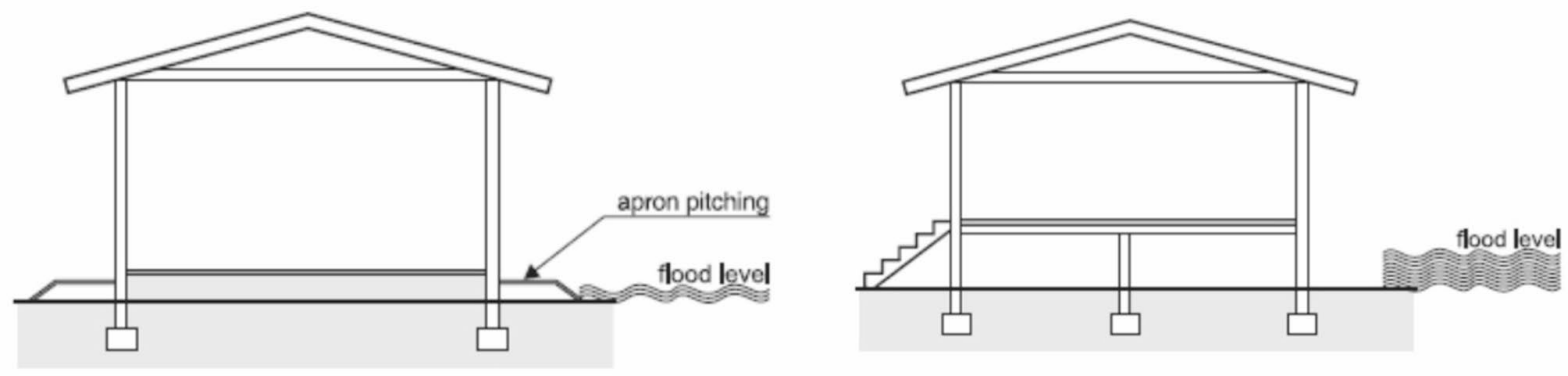
Protection measures against inundation effects. (Source: BMTPC).

**Fig. 24:**
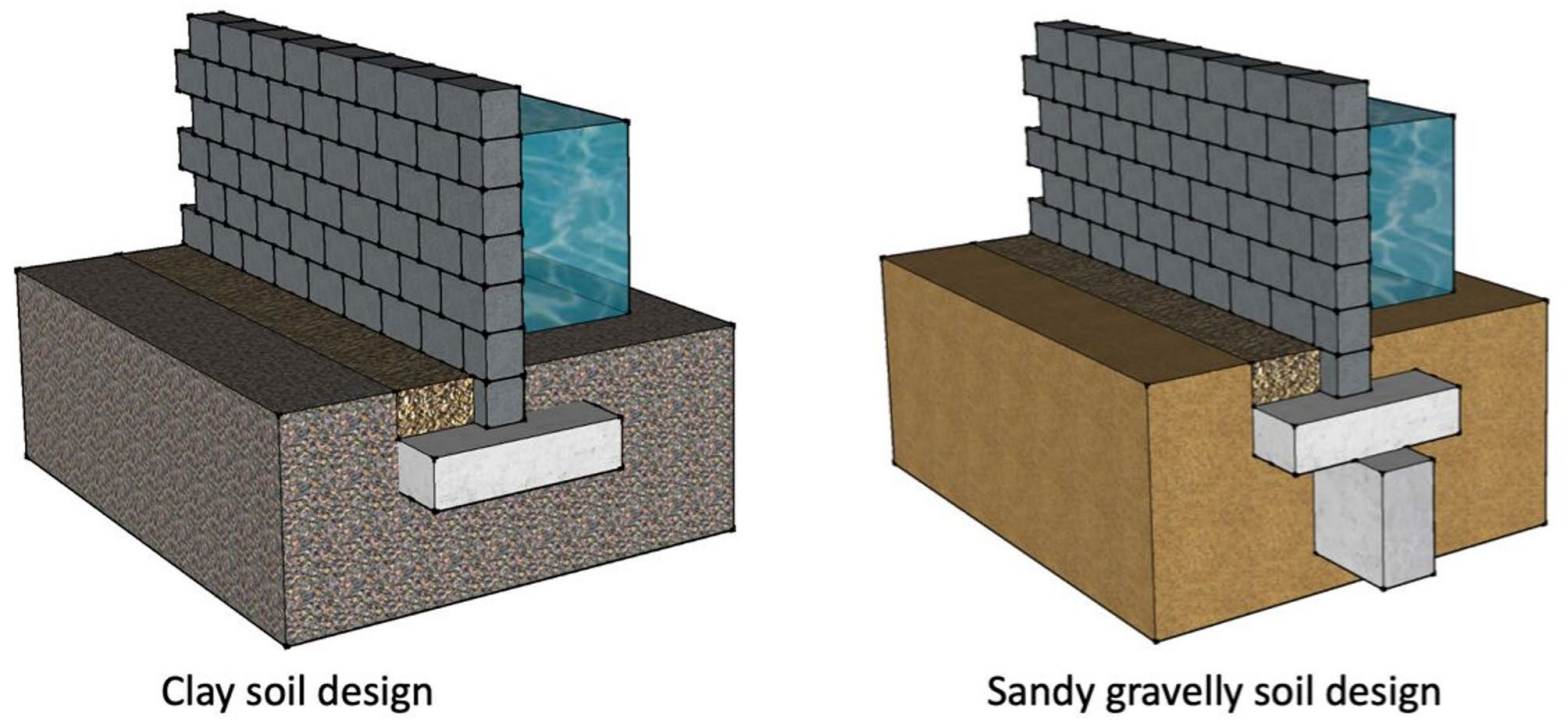
Flood Defence walls in flood prone areas. (Source: Retaining wall solutions page).

**Fig. 25:**
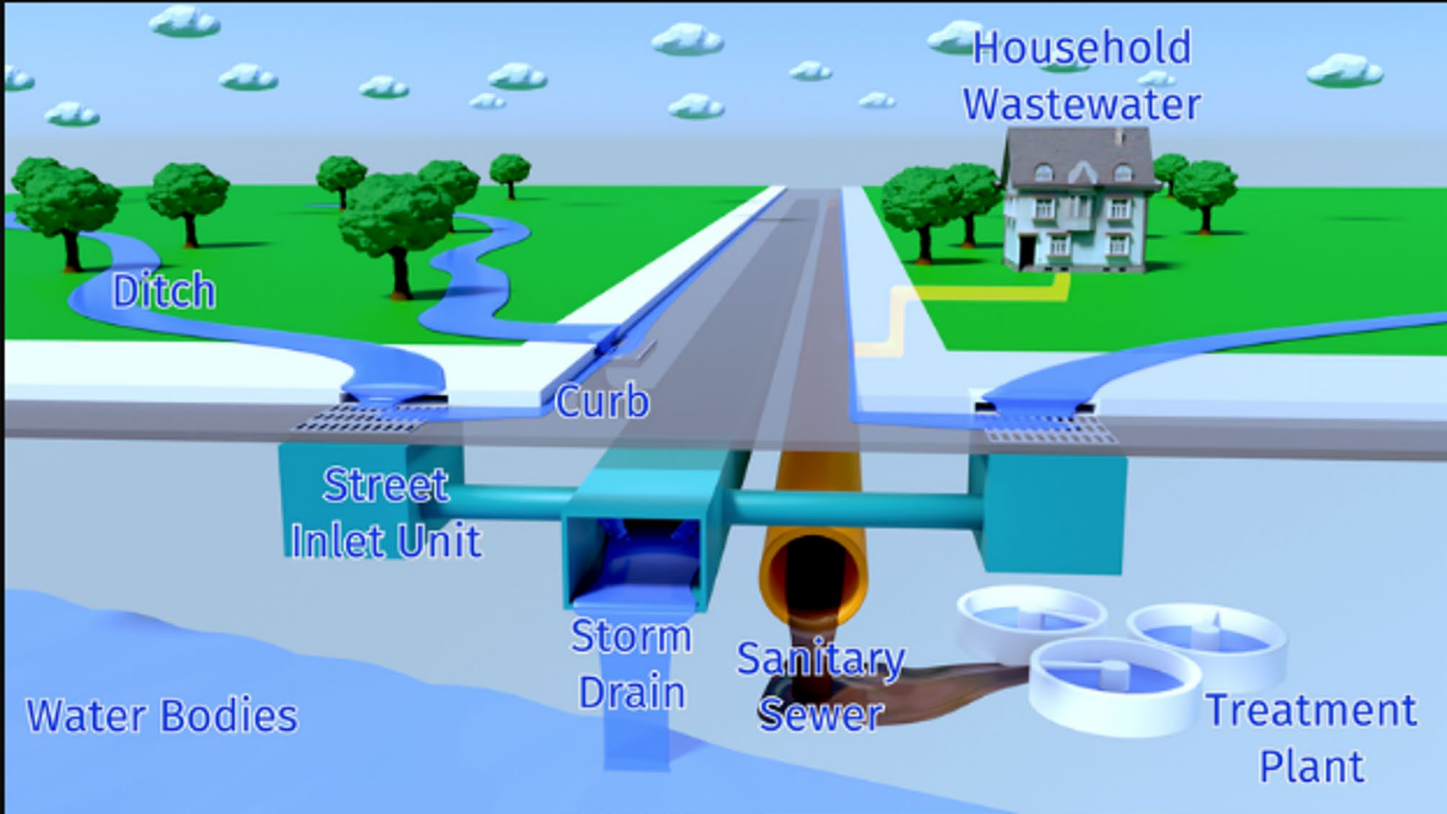
Predicted urban drainage systems.

**Fig. 26:**
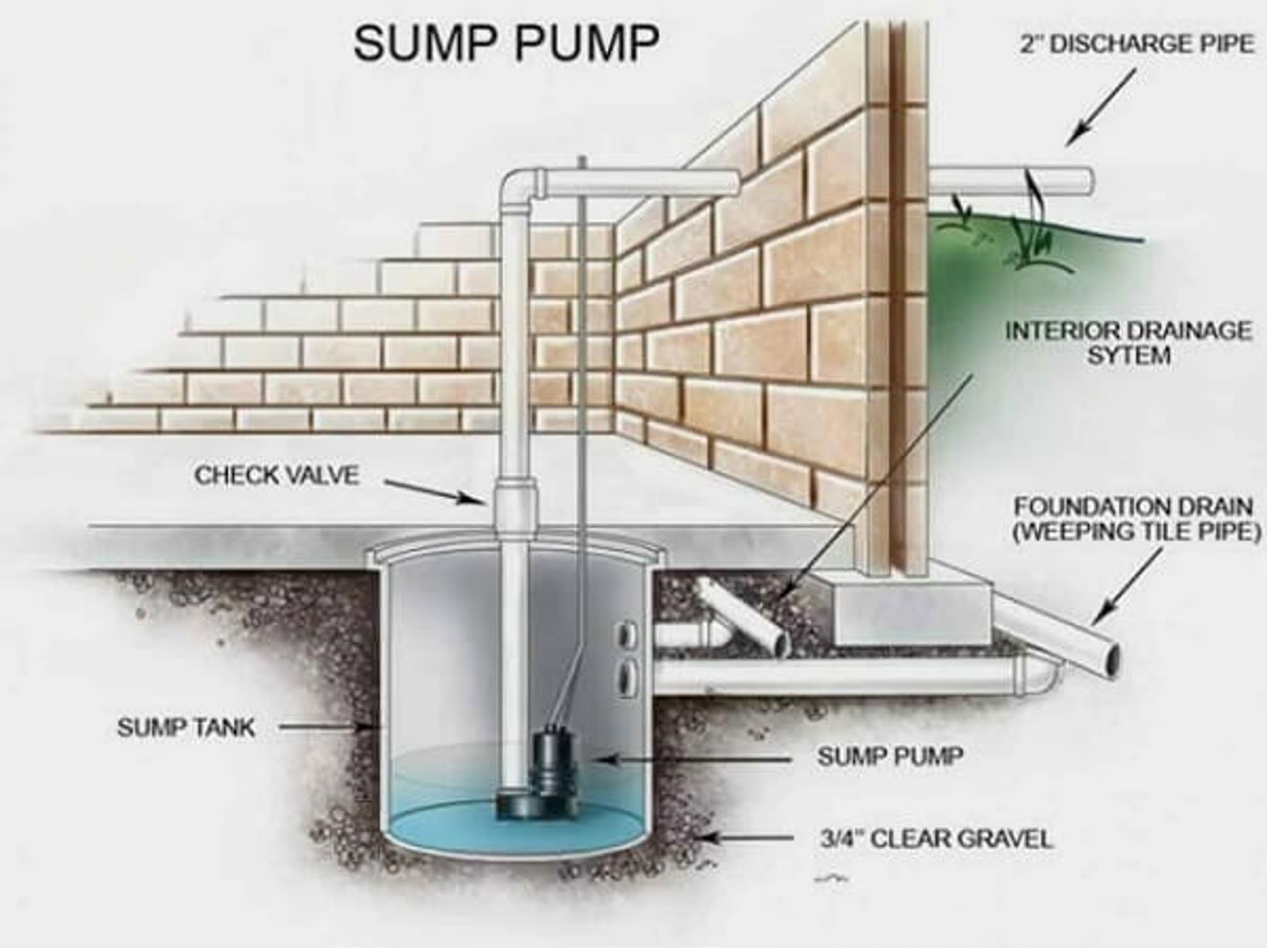
Underground storage of flood water using Sump pump.

**Fig. 27:**
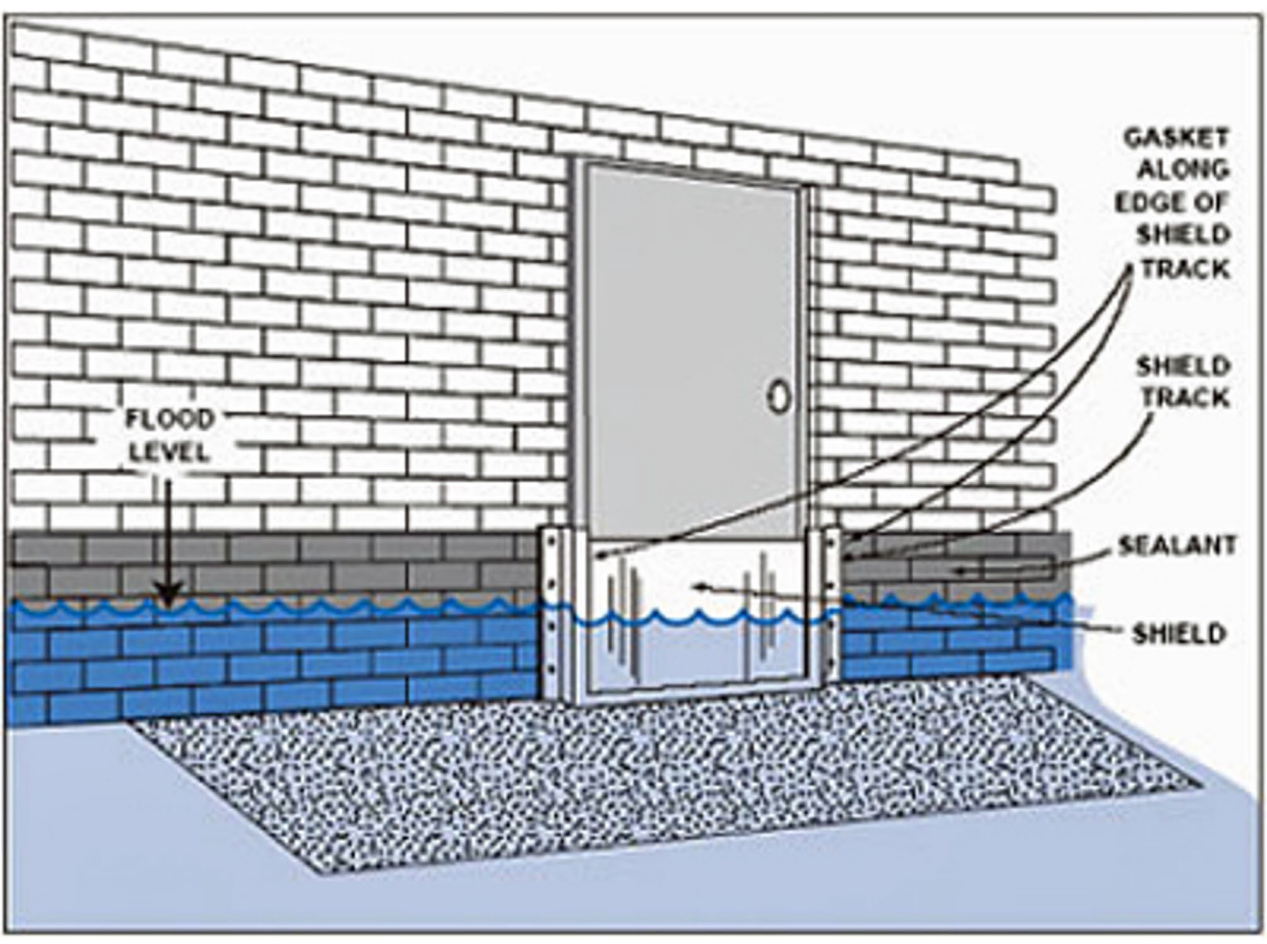
Flood resistant materials to resist the floods from entering inside the houses.

In Hyderabad, like in many other flood-prone regions, implementing effective protection measures for buildings is crucial to mitigate flood-related damages. Some key measures include:

- Protecting Structures: Guidelines of “Improving flood resistance of housing” submitted by Building Materials & Technology council Ministry of Housing & Urban Poverty Alleviation Government of India, New Delhi (BMTPC) given provisions for the buildings in flood prone areas. Construction regulations may mandate plinth level of building should be 0.6 m above flood submersion levels under the mean annual flood. Figure 23 shows the earth mound and raised structure to protect buildings against inundation.
- Flood Barriers and Walls: Installing flood barriers or walls around buildings can prevent water from entering the premises during floods. Most defence walls less than 2 m with wall height from 450 mm to 2025 mm (Retaining wall Solutions page). Figure 24 shows the clay soil design and sandy gravel design for flood defence wall.
- Improved Drainage Systems: Ensuring proper drainage systems, including gutters, drains, and culverts, can help divert floodwaters away from buildings and reduce the risk of inundation. Figure 25 is taken from^7^, worked on urban stormwater networks.
- Green Infrastructure: Incorporating green infrastructure elements like rain gardens and permeable pavements can help absorb and manage excess water during heavy rainfall, reducing flood risks.
- Floodproofing: Implementing floodproofing measures, such as sealing walls, installing backflow valves, and waterproofing basements, can help prevent water infiltration into buildings.
- Sump Pumps: Installing sump pumps in basements or low-lying areas can help remove excess water and prevent flooding inside buildings as shown in Fig. 26.
- Flood-Resistant Materials: Using flood-resistant building materials for construction, such as concrete, brick, and water-resistant coatings, can minimize flood damage to structures as shown in Fig. 27.

## Conclusions

Floods pose significant risks to buildings in urban areas like Hyderabad, where multiple factors contribute to their occurrence. To assess the vulnerability of structures, an investigation is made on specific areas between Gandipet and Narsingi, utilizing tools such as HEC-RAS, QGIS, Arc-GIS and XGBoost. The following conclusions are drawn from the present study:

- The Discharge data, Metrological data is collected for Gandipet Guage Station and the required shapefile layers of Buildings and DEM of study area is collected.
- The Discharge data and DEM is used for 2D unsteady flow simulation in HECRAS software to compute flood depth map, flood velocity map and water surface elevation maps of downstream section of Osman Sagar in the RAS-Mapper.
- HEC-Ras software is used as a tool to generate the Flood inundation map after the simulation.
- The performance of 2D HEC-RAS model is evaluated through comparison between point-based depth of water from model output to the real time data of water level.
- The RMSE value which is 0.01 m higher than MAD validates the model capability to replicate the spatial distribution of inundation.
- The generated Flood inundation map is overlaid with the building shape file layer in QGIS to identify the flood prone areas and number of buildings that can submerge due to the flood.
- XGBOOST algorithm is used to predict future floods by developing flood prediction graph in this research. These tools offer valuable insights into flood patterns, enabling informed decisions on protective measures for buildings.
- This FVI is a degree indicating the area susceptible to flood which indicates the vulnerability of exposed elements using different types of influencing indicators.
- The application of the XGBoost model for flood level prediction demonstrated high accuracy with an R^2^ of 0.945, RMSE of 0.795 mm, and MAE of 0.514 mm.
- The XGBoost model results confirm the reliability and effectiveness of the model in predicting flood levels based on atmospheric variables.
- The normalization technique used for calculating indicator value between 0 and 1 based upon its direct or inverse relation with the flood vulnerability.
- The resultant value of FVI indicates that the selected region is not most vulnerable but still it faces significant flood risk.
- The sensitivity analysis of FVI reveals that the socio-economic indicators like population density, literacy rate, and infrastructure, exhibit the highest sensitivity in the study area where population density and infrastructure development are substantially higher than in other districts.
- Elevation, slope, Drainage density, LULC and Rainfall maps are used to generate flood susceptibility map by applying weights based upon its degree of influencing the occurrence of flood.
- Flood hazard map segregates the regions based on intensity of risk level from 1 to 5. The generated flood susceptibility map indicates that more than 65% of the Osmansagar basin is vulnerable to flooding.
- In flood hazard map, by incorporating the latest datasets of rainfall and LULC, the analysis accurately represents the present-day conditions that enhances the reliability and relevance of the flood hazard and risk mapping outcomes.
- The sensitivity analysis highlights that Annual rainfall and LULC are the most critical factors affects flood risk mapping.

From the present study the future recommendations are listed below:

- Key strategies are identified for safeguarding buildings from floods include elevating structures above flood levels to reduce the risk of inundation. Installing flood barriers and walls around buildings effectively prevents water from entering premises during flood events.
- Improved drainage systems, including gutters, drains, and culverts, play a crucial role in diverting floodwaters away from buildings, thus minimizing the risk of inundation and water damage.
- Incorporating sump pumps in basements or low-lying areas helps remove excess water and prevent interior flooding. The use of flood-resistant materials like concrete, brick, and water-resistant coatings further mitigates flood-related damages to structures.
- Floodproofing measures such as sealing walls, installing backflow valves, and waterproofing basements offer additional protection by preventing water infiltration into buildings. Green infrastructure elements such as rain gardens and permeable pavements can absorb and manage excess water, thereby reducing flood risks in urban areas.
- In conclusion, through the utilization of advanced tools and the implementation of comprehensive protection measures, the vulnerability of buildings to floods in cities like Hyderabad can be significantly reduced. Proactive planning, informed decision-making, and community engagement are vital in enhancing the resilience of buildings and communities, ensuring their safety and well-being in the face of flood-related challenges.

As the present study focuses on urban flood prediction and vulnerability assessment using machine learning, hydrodynamic modeling and spatial analysis, future work can be extended to incorporate geomorphological factors such as soil aggradation and degradation which plays a key role in altering land surface characteristics that influences flood extent and severity. This integration would provide a more holistic understanding on flood risk.

## Data Availability

All the data used in this study is available from the corresponding author upon request.
